# Ultrasound‐Activatable Piezoelectric Hydrogel Reprograms Mitochondrial Epigenetics for Osteoarthritis Therapy via the mTOR/GATD3A Axis

**DOI:** 10.1002/advs.76140

**Published:** 2026-06-16

**Authors:** Hui Zheng, Pengfei Yan, Peng Liu, Chang Yan, Mengqi Zhao, Zuyong Wang, Chao Ma, Rongkai Zhang, Xi Cui, Swee Hin Teoh

**Affiliations:** ^1^ College of Materials Science and Engineering Hunan University P. R. China; ^2^ Department of Joint Surgery Center for Orthopaedic Surgery The Third Affiliated Hospital of Southern Medical University (Academy of Orthopedics, Guangdong Province) Guangzhou Guangdong P. R. China; ^3^ Tsinghua University Beijing P. R. China

**Keywords:** epigenetic regulation, GATD3A, mitophagy, mTOR, osteoarthritis, piezoelectric hydrogel

## Abstract

The avascular nature of cartilage hinders drug delivery for osteoarthritis (OA) therapy. We engineered a biomimetic piezoelectric hydrogel (CMB Gel) by embedding chondrocyte membrane‐camouflaged, CAP peptide‐grafted barium titanate nanoparticles into a dynamic borate ester‐crosslinked network, enabling active cartilage targeting and on‐demand ultrasound activation. Under ultrasound, the piezoelectric component generates local electrical signals, triggering Ca^2^^+^ influx via voltage‐gated calcium channels and AMPK activation. Activated AMPK inhibits the mTOR pathway, inducing epigenetic reprogramming via H3K27 acetylation at the GATD3A promoter. Upregulated GATD3A stabilizes TFAM and enhances PINK1/Parkin‐mediated mitophagy, clearing damaged mitochondria and reducing oxidative stress in chondrocytes. In a mouse destabilization of medial meniscus model, ultrasound‐activated CMB Gel attenuated cartilage degradation, osteophyte formation, and synovitis. Its therapeutic efficacy was validated in human OA cartilage explants. This work presents a multifunctional targeted delivery platform that converts mechanical energy into epigenetic signals to restore cellular homeostasis, offering a promising strategy for OA and other mechanosensitive degenerative diseases.

## Graphical abstract

An ultrasound‐activatable piezoelectric hydrogel reprograms chondrocyte mitochondrial epigenetics via the mTOR/GATD3A axis, clearing damaged mitochondria and alleviating osteoarthritis progression in both mouse models and human cartilage explants.

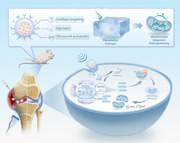

## 1 Introduction

Osteoarthritis (OA) is a common degenerative joint disease characterized by the progressive degeneration of articular cartilage and the destruction of the extracellular matrix (ECM), resulting in chronic pain, inflammation, and functional impairment of the affected joints [1, 2, 3]. Chondrocyte senescence is closely associated with mitochondrial dysfunction, which often leads to oxidative stress and accumulation of damaged mitochondria, thereby accelerating cartilage destruction. Therefore, maintaining mitochondrial health is of vital importance for the treatment of osteoarthritis [4, 5, 6, 7, 8, 9].

A range of interventions is available for managing symptoms in patients with OA. However, no disease‐modifying therapies are currently accessible. Several biologics and small molecules, such as synthetic growth factors, matrix‐degrading enzyme inhibitors, and anti‐inflammatory agents, have shown promise in inhibiting cartilage degeneration in preclinical studies. Unfortunately, these candidates have failed to progress from clinical trials into clinical practice due to their limited patient benefits. A major challenge in the pharmacological treatment of OA is maintaining effective drug concentrations around chondrocytes. The avascular nature of articular cartilage results in insufficient biodistribution following systemic administration. Direct intra‐articular injection represents a rational approach to enhance joint bioavailability and minimize systemic side effects [10]. Nevertheless, even local injection proves insufficient, as drugs are rapidly cleared from the joint cavity via synovial capillary and lymphatic drainage, with a short residence time of merely 1–4 h [11]. The small fraction of drug remaining intra‐articularly is largely excluded from chondrocytes by the dense cartilage ECM. The cartilage ECM, synthesized by chondrocytes, is a dense network composed of collagen fibers, proteoglycans, and numerous other macromolecules. Consequently, the ECM not only protects chondrocytes but also poses a substantial steric barrier, hindering the penetration of therapeutic molecules from the synovial fluid and their subsequent internalization by chondrocytes. Coupled with the clinical need to minimize infection risks associated with repeated intra‐articular injections, the twin biological hurdles of short drug half‐life in the synovial fluid and inadequate diffusion into the cartilage severely restrict the potential for sustained therapy, thereby compromising long‐term outcomes [12]. Therefore, an ideal drug delivery platform for OA should be capable of adequately penetrating the cartilage before clearance, binding to sites within it, and thereby transforming this barrier into a drug reservoir for sustained intra‐articular release.

To achieve efficient cartilage targeting, we employed a biomimetic strategy by coating nanoparticles with chondrocyte membrane (CM). The CM coating provides homologous targeting capability, leveraging surface adhesion molecules such as cadherins and integrins to enable nanoparticles to preferentially bind to and be internalized by chondrocytes [13]. This approach has been shown to reduce nanoparticle clearance and increase accumulation at the cartilage site. On this basis, we further grafted CAP targeting peptides onto the CM coated barium titanate (BTO) nanoparticles to enhance their affinity for the cartilage ECM. The CAP peptide (sequence: DWRVIIPPRPSA‐C) is a cartilage‐binding peptide that specifically targets collagen VI, which is abundantly expressed in the extracellular matrix of articular cartilage. Conjugation of this peptide to the nanoparticle surface via a PEG linker (CLS‐PEG‐CAP) enhances the affinity of the nanoparticles to the cartilage matrix, thereby improving their retention and cellular uptake.

In recent years, piezoelectric materials have attracted considerable attention due to their ability to generate local electrical signals under mechanical stimulation [14]. This feature can be harnessed to regulate various biological processes, including mitochondrial function and autophagy [15, 16]. Ultrasound, as a highly controllable and tissue‐penetrating external stimulus, has been proven to effectively activate piezoelectric materials, providing a new strategy for achieving targeted therapy. By combining ultrasound‐activated piezoelectric nanomaterials with a biomimetic targeting system, we have proposed a novel OA treatment strategy. This strategy not only enables precise drug delivery to damaged cartilage but also utilizes mechanical energy to activate mitochondrial autophagy, a key process for maintaining cellular homeostasis.

**FIGURE 1:**
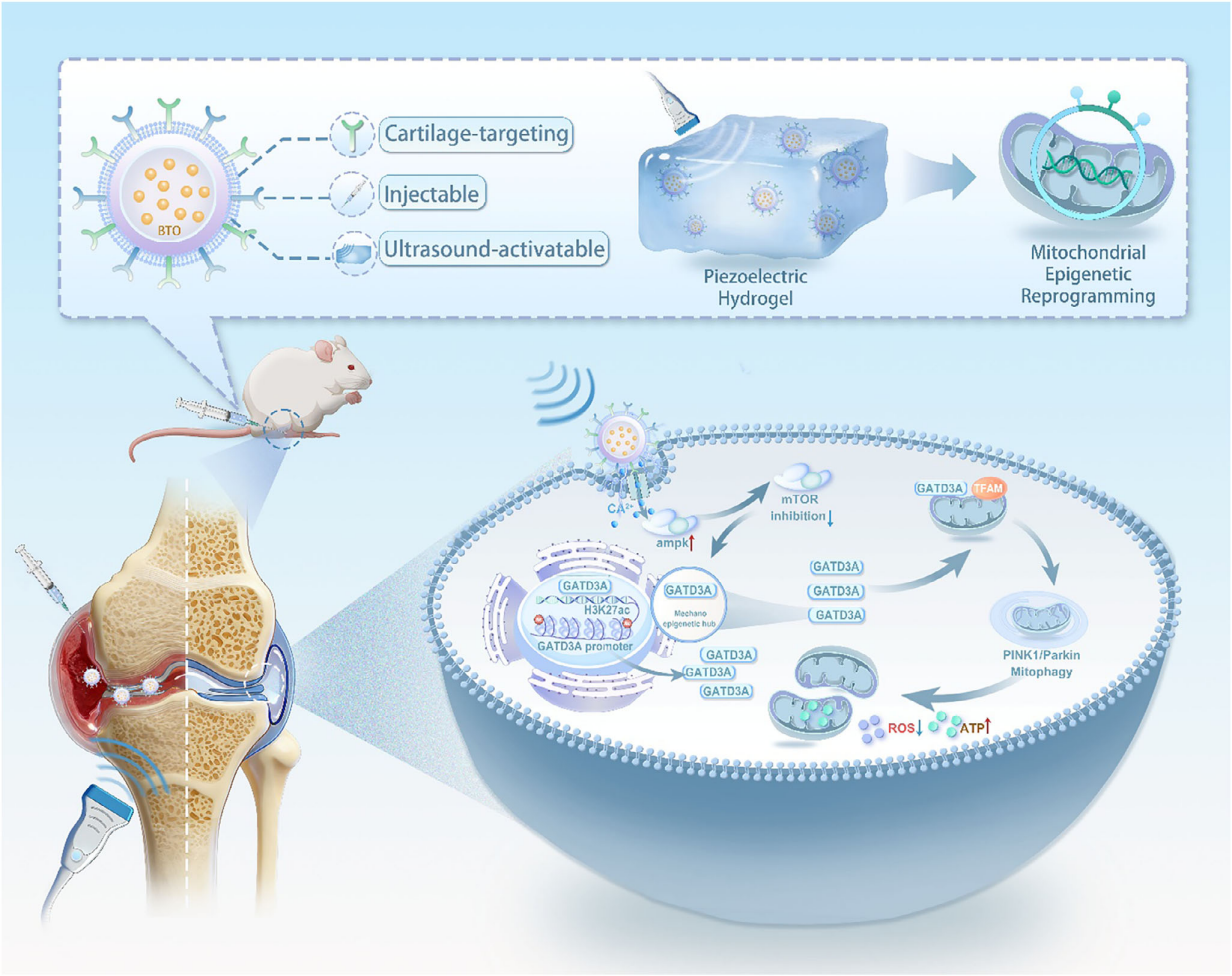
The design strategy of biomimetic piezoelectric hydrogel and the schematic diagram of the mechanism of cartilage regeneration.

In this study, we report a biodegradable biomimetic piezoelectric hydrogel, which achieves excellent chondrocyte targeting, permeation, and retention properties by embedding barium titanate (BTO) coated with chondrocyte membrane and grafted with CAP targeting peptides into a dynamic borate ester cross‐linked network composed of chondroitin sulfate and collagen. Under ultrasound activation, the hydrogel generates local piezoelectric signals, inhibits the mTOR pathway, and subsequently induces epigenetic reprogramming by enhancing H3K27 acetylation in the GATD3A promoter region. The upregulation of GATD3A further stabilizes TFAM and activates the PINK1/Parkin‐mediated mitochondrial autophagy pathway, thereby eliminating damaged mitochondria, alleviating oxidative stress, and restoring chondrocyte function. In IL‐1β induced chondrocyte models and mouse DMM models, this hydrogel system demonstrates significant chondrocyte protection and beneficial effects (Figure 1 and Figure S1). This study not only provides an efficient chondrocyte‐targeted delivery platform, but also pioneers a new paradigm for OA treatment through “mechanical electrical signal” regulation of autophagy and epigenetics, laying a solid foundation for the development of precise treatment strategies for OA and other mechanically sensitive degenerative diseases.

## 2 Results

### 2.1 Synthesis and Structural Characterization of the Injectable Biomimetic Hydrogel

**FIGURE 2:**
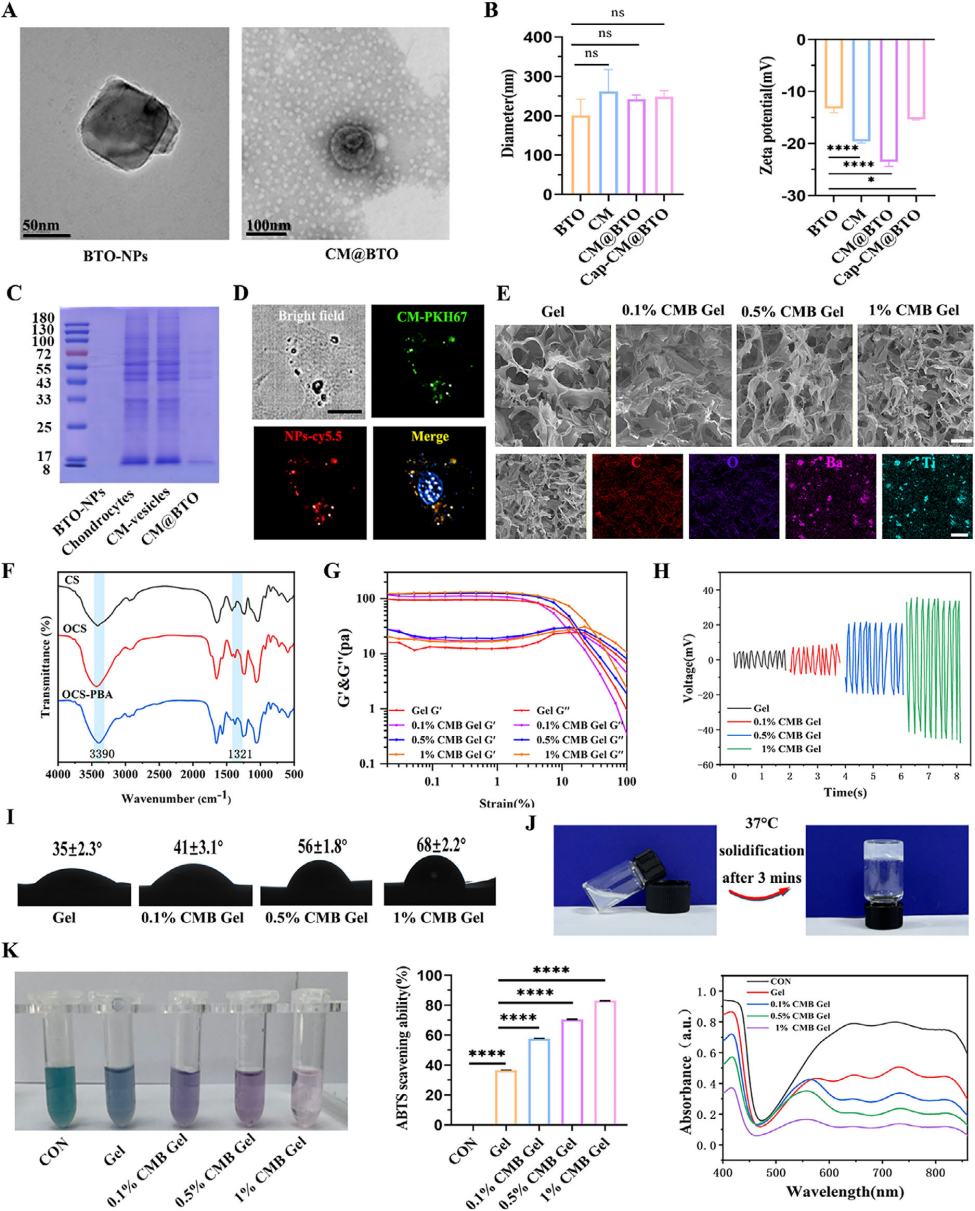
Preparation and Characterization of Biomimetic Nanoparticles. (A) Transmission electron microscopy images of BTO‐NPs and CM@NPs. Scale bar, 100 nm. (B) Mean hydrodynamic diameters and surface zeta potentials of BTO‐NPs, CM vesicles, CM@BTO, and Cap‐CM@BTO measured by dynamic light scattering (DLS) (n = 3 for each group). (C) Protein expression profiles of BTO‐NPs, fresh mouse chondrocytes, CM vesicles, and CM@NPs separated by sodium dodecyl sulfate‐polyacrylamide gel electrophoresis (SDS‐PAGE) and subsequently stained with Coomassie blue. (D) Representative confocal microscopy images of mouse chondrocytes treated with CM@NPs. The BTO core was labeled with Cy5.5 (red), the CM shell with PKH67 (green), and the nuclei with DAPI (blue). Scale bar, 20 µm. (E) SEM images of the internal structures of different hydrogel scaffolds. Scale bar, 100 µm. (F) Fourier transform infrared spectra of CS, OCS, and OCS‐PBA hydrogels. (G) Rheological properties of the hydrogels. G' represents the storage modulus. G'' represents the loss modulus. (H) Output voltages of different hydrogels. (I) Water contact angles of different hydrogel scaffolds. (J) Piezoelectric hydrogels form rapidly (~ 3 min) at physiological temperatures. (K) UV–vis detection of ATBS radical scavenging capacity of hydrogel scaffolds after treatment, with solution color changes and quantitative analysis. Statistical analysis was performed using one‐way analysis of variance (ANOVA) combined with Tukey's post hoc test. n = 3, ^*^ *p* < 0.05, ^***^ *p* < 0.001. ns, no significant difference.

Primary mouse chondrocytes were isolated, and their CM were extracted following the commercial kit's instructions [17]. The CM vesicles were subsequently coated onto BTO nanoparticle cores by repeatedly extruding the mixture through a polycarbonate membrane with 400 nm pores [18]. Transmission electron microscopy (TEM) images revealed a uniform BTO core (50.14 ± 4.67 nm) surrounded by an outer layer of CM, indicating successful coating (Figure 2A). Dynamic light scattering (DLS) analysis showed that the average hydrodynamic diameter of CM@BTO (242.133 ± 0.12 nm) was slightly larger than that of uncoated BTO nanoparticles (200.367 ± 3.28 nm). The surface zeta potential of CM@BTO (‐24.40 ± 0.30 mV) was comparable to that of CM vesicles alone (‐20.03 ± 0.52 mV) but significantly more negative than that of the uncoated BTO core (‐12.21 ± 0.21 mV; *p* < 0.0001) (Figures 2B and S2). Both TEM and DLS data confirmed the successful encapsulation of BTO cores by the CM. Following functionalization with CLS‐PEG‐CAP, the diameter increased from 242.133 ± 0.12 nm (CM@BTO) to 248.167 ± 036 nm (Cap‐CM@BTO). The zeta potential also shifted from ‐24.40 ± 0.30 mV (CM@BTO) to ‐15 ± 2 mV (Cap‐CM@BTO), confirming the successful grafting of the targeting peptide. To verify the retention of membrane proteins during the coating process, CM@BTO nanoparticles were first analyzed by SDS‐PAGE with Coomassie Blue staining (Figure 2C). The protein profile of CM@BTO closely resembled that of the native CM, indicating successful preservation of CM proteins. The coating was further validated using a dual fluorescence labeling approach. Confocal microscopy results demonstrated that the Cy5.5 labeled BTO core (red) was effectively encapsulated by the PKH67 labeled CM (green), showing clear co‐localization (yellow) in the cytoplasm after cellular internalization by chondrocytes (Figure 2D and Figure S3). The microstructure and morphology of the different hydrogel scaffolds are shown in Figure 2E. All scaffolds exhibited an irregular but interconnected porous structure, which is beneficial for the transport of nutrients and metabolites [19]. EDS mapping confirmed the homogeneous distribution of Ba and Ti elements throughout the hydrogel scaffold, indicating the successful and non‐aggregated incorporation of Cap‐CM@BTO—a crucial improvement over the typical aggregation tendency of BTO nanoparticles. Fourier‐transform infrared (FT‐IR) spectroscopy was employed to confirm hydrogel formation (Figures 2F and S4). The spectrum of CS showed a characteristic broad absorption peak at 3390 cm^−^^1^, corresponding to O‐H stretching vibrations. The spectrum of the modified OCS‐PBA additionally displayed the characteristic peak of the borate ester bond at 1321 cm^−^^1^, confirming the successful synthesis of the hydrogel network [20].

Rheological measurements (Figure 2G) demonstrated that the storage modulus (G') of the hydrogel scaffolds increased with higher Cap‐CM@BTO content. These results indicate that the incorporation of Cap‐CM@BTO nanoparticles stabilized the microstructure of the Col hydrogel scaffold via a complementary crosslinking mechanism. While OCS‐PBA formed covalent crosslinks between collagen chains, the Cap‐CM@BTO nanoparticles provided additional crosslinking sites via their surface functional groups. This synergistic interaction created an interconnected network structure that effectively enhanced the mechanical properties, thereby addressing the inherent mechanical weakness of pure collagen hydrogel scaffolds [21]. The output voltage of the hydrogels was subsequently evaluated (Figure 2H). Incorporating Cap‐CM@BTO into the hydrogel scaffold significantly enhanced the output voltage. As the Cap‐CM@BTO ratio increased from 0.1% to 1% (w/v), the output voltage of the piezoelectric hydrogel scaffolds rose from 10.12 to 30.25 mV. Furthermore, the 1% Cap‐CM@BTO/Gel (CMB Gel) hydrogel scaffold exhibited a d_33_ value of 21.6 pC/N, which was significantly higher than that of other hydrogel scaffolds (Figure S5). This enhanced mechanoelectrical conversion originates from efficient stress transfer between the collagen matrix and the uniformly dispersed Cap‐CM@BTO nanoparticles. Under ultrasound (US) stimulation, the aligned dipole moments within the Cap‐CM@BTO nanoparticles generate a synergistic polarization effect, amplifying the overall piezoelectric response. The resulting self‐powered system demonstrates a clinically feasible electrical output without requiring implanted electrodes, highlighting its potential for noninvasive therapeutic applications [22]. The water contact angle of the hydrogels increased from 35° to 68° as the Cap‐CM@BTO content increased from 0% to 1% (w/v) (Figure 2I). This increased hydrophobicity is attributed to the chemical composition; the chondrocyte membrane layer coating the BTO nanoparticles contains phospholipid hydrophobic tails. As the BTO content increases, more of these hydrophobic chains are exposed on the surface, leading to a larger contact angle. The moderate hydrophobicity (68°) achieved in the 1% (w/v) CMB Gel group offers a dual advantage: it enhances cartilage adhesion via the Cassie‐state air cushion effect [23], while remaining at a level that does not compromise cytocompatibility. By optimizing the Cap‐CM@BTO concentration at 1% (w/v), this study achieved an optimal balance between adhesive performance and biosafety. The surface functional groups of the nanoparticles facilitated further crosslinking, resulting in the formation of a dual‐network structure at 37°C (Figure 2J). The injectability of hydrogels is of vital importance for minimally invasive surgeries in the treatment of osteoarthritis. Figure S6 demonstrates this injectability. The in vitro antioxidant capacity of the hydrogels was evaluated using an ABTS radical scavenging assay. The results showed that the hydrogels possessed inherent antioxidant activity due to the hydroxyl groups of chondroitin sulfate, manifested by a decrease in absorbance at 723 nm. This antioxidant performance was further significantly enhanced after the introduction of Cap‐CM@BTO (Figure 2K), achieving a scavenging rate of 83.11% accompanied by a visible lightening of the solution color. This suggests that the piezoelectric component may synergistically enhance antioxidant efficacy by promoting electron transfer and increasing contact between radicals and active sites. In summary, the incorporation of Cap‐CM@BTO endowed the hydrogel with both desirable piezoelectric responsiveness and efficient ROS scavenging capability, providing a critical functional foundation for its application in OA therapy.

The enzymatic degradation resistance of the hydrogels was assessed by immersing samples in a type I collagenase solution and observing morphological changes over time (Figure S7). The Gel group was almost completely degraded within 21 days, whereas the CMB Gel scaffold exhibited significantly higher weight retention. In vivo degradation studies further confirmed that the hydrogel scaffold maintained a high retention rate at day 21(Figure S8). To further evaluate the actual residence time of the hydrogels in the joint cavity, we next performed in vivo retention tracking using Cy5.5 labeled Gel and CMB Gel. After intra‐articular injection into the mouse knee joints, fluorescence signals were monitored by IVIS imaging at days 1, 3, 5, and 7, post‐injection (Figure S9). Consistent with its slower degradation, CMB Gel exhibited a significantly prolonged retention time, with detectable signal up to day 7, whereas the Gel signal was nearly absent by day 5. This enhanced stability is attributed to the covalent crosslinks between OCS‐PBA and collagen chains, which reinforce the hydrogel's network structure. The increased crosslinking density impedes enzyme penetration and proteolytic cleavage, thereby reducing the accessibility of hydrolytic sites within the collagen matrix to collagenase [24].

### 2.2 Cartilage Adhesion, Penetration, Retention, and Targeting

**FIGURE 3:**
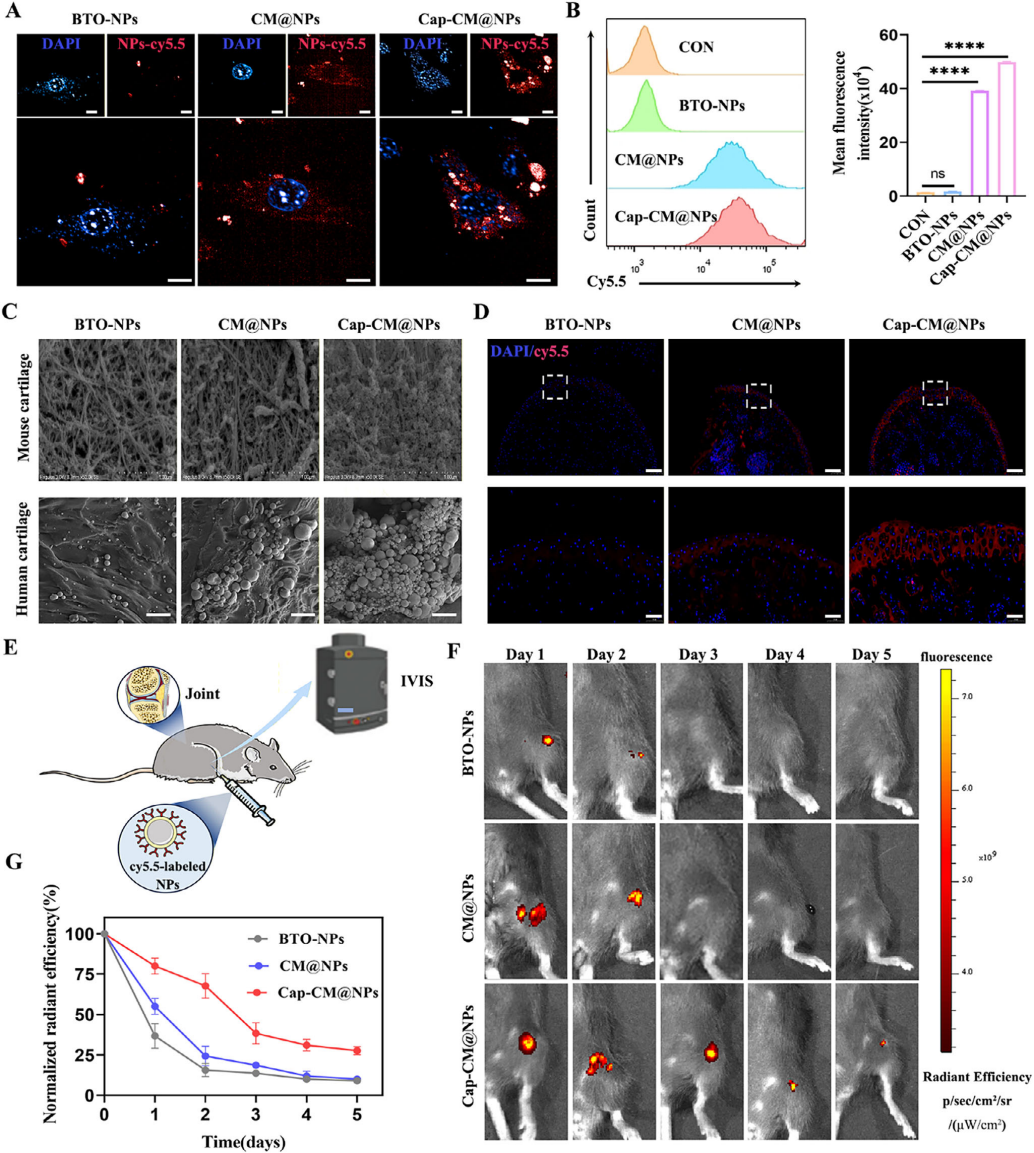
CM‐NPs prolong the retention time of the joint through cartilage adhesion and penetration. (A) Representative confocal microscope images of mouse chondrocytes after incubation with BTO‐NPs, CM@NPs, and Cap‐CM@NPs (200 µg/mL) for 24 h. The nanoparticles were labeled with Cy5.5 (red). Scale bar,50 µm. (B) Flow cytometry analysis and corresponding quantitative mean fluorescence intensity after incubation with BTO‐NPs, CM@NPs, and Cap‐CM@NPs (200 µg/mL) for 1 h. (C) Representative scanning electron microscope images of the tissue distribution and adhesion of BTO‐NPs, CM@NPs, and Cap‐CM@NPs on the surface of mouse femoral heads cartilage. Representative scanning electron microscope images of the tissue distribution and adhesion of BTO‐NPs, CM@NPs, and Cap‐CM@NPs on the surface of human femoral condyles cartilage. (D) Representative confocal microscope image showing the cross‐section of mouse cartilage sections after 3 days of incubation. Bottom: enlarged image of the white box area. Nanoparticles (red, Cy5.5); cell nucleus (blue, DAPI). Scale bar,50 µm. (E) Schematic diagram of IVIS assessment. (F) Representative IVIS images of C57BL/6 mice 5 days after a single intra‐articular injection of free BTO‐NPs, CM@NPs, and Cap‐CM@NPs. (G) Quantitative analysis of the standardized fluorescence radiation efficiency over the 5 ‐day time course in the mouse knee joint (n = 3). Statistical analysis was performed using one‐way analysis of variance (ANOVA) combined with Tukey's post hoc test. ^*^ *p* < 0.05, ^***^ *p* < 0.001. ns, no significant difference.

We first investigated the effect of the Cap‐modified cell membrane coating on the efficiency of nanoparticle uptake by chondrocytes, the biologically relevant target cells in OA. Chondrocytes treated with Cy5.5‐labeled Cap‐CM@NPs exhibited strong red fluorescence (Figure 3A). In contrast, chondrocytes incubated with unmodified BTO‐NPs showed weak fluorescence, indicating the critical importance of the Cap‐modified membrane coating for chondrocyte targeting. Consistent with this observation, quantitative flow cytometry analysis revealed a significantly higher mean fluorescence intensity in chondrocytes after incubation with Cy5.5 labeled Cap‐CM@NPs compared to those treated with BTO‐NPs or CM@NPs (Figure 3B). Considering that nanoparticles must diffuse through the cartilage ECM before being internalized by chondrocytes, we next examined the interaction between nanoparticles and the cartilage ECM. We utilized mouse cartilage explants with their native 3D ECM structure to study the penetration of Cap‐CM@NPs into cartilage. Initially, the nanoscale interaction between nanoparticles and the cartilage ECM was investigated. After 24 h of incubation with Cap‐CM@NPs, followed by phosphate‐buffered saline (PBS) washing to simulate synovial fluid clearance, SEM images showed a substantial number of Cap‐CM@NPs adsorbed onto the mouse cartilage fibrous network (Figure 3C). In contrast, minimal nanoparticle adhesion was observed with BTO‐NPs, confirming that the Cap modified membrane coating confers superior adhesion capability to the cartilage ECM.

Given that cartilage thickness and the size of fibrous pores vary with animal size, we further investigated nanoparticle attachment in cartilage samples from osteoarthritis patients. Compared to mouse cartilage, the Cap‐CM@NPs group demonstrated the most effective attachment in degenerated human cartilage (Figure 3C). Cartilage degeneration did not impede the binding of Cap‐CM@NPs to cartilage fibers, highlighting the advantage of the Cap peptide for cartilage binding. Subsequently, mouse cartilage explants were incubated with Cy5.5 labeled nanoparticles for 3 days, sectioned, and examined by confocal microscopy. Compared to the non‐targeting control (BTO), the fluorescence intensity in the superficial zone of cartilage was 3.2 times higher for Cap‐CM@NPs (Figure 3D). An in vivo imaging system was used to examine the retention of Cy5.5‐labeled nanoparticles within the mouse joint cavity over a 5‐day period (Figure 3E). Following a single intra‐articular injection, the fluorescence signal in the Cap‐CM@NPs group was significantly stronger than that in the other three groups at all time points (Figure 3F); a pronounced signal remained even on day 5 post‐injection. The standardized radiant efficiency within the dissected joint region was quantified (Figure 3G). These results collectively confirm that the targeting peptide‐modified cell membrane coating successfully enables specific nanoparticle migration to cartilage and prolongs their retention within the joint.

### 2.3 Biocompatibility of the Piezoelectric Hydrogel

**FIGURE 4:**
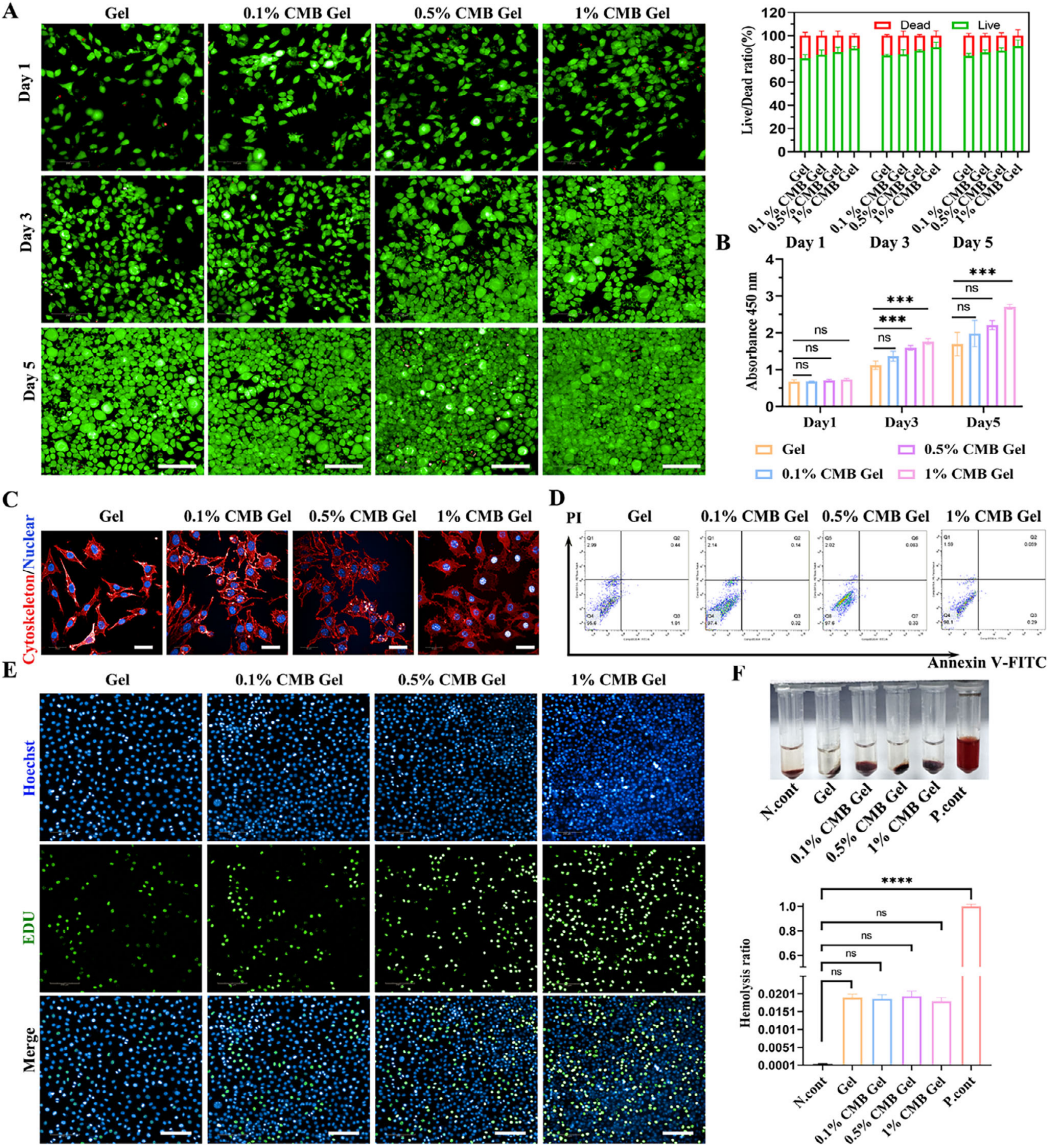
Evaluation of the biocompatibility of the hydrogel. (A) The CLSM images show the live/dead staining of L929 cells on different hydrogels on days 1, 3, and 5. Scale bar,200 µm. (B) The proliferation of L929 fibroblasts cultured on different hydrogels was studied using the CCK‐8 assay. (C) The cytoskeleton staining of L929 cells on various hydrogels is shown in the CLSM images. Scale bar,50 µm. (D) The apoptosis of L929 cells cultured on different hydrogels was detected by flow cytometry. (E) The EdU staining of L929 cells on various hydrogels is shown in the CLSM images. Scale bar,200 µm. (F) Quantitative analysis of the hemolysis rate of the samples. Statistical analysis was performed using one‐way analysis of variance (ANOVA) combined with Tukey's post hoc test. n = 5, ^*^ *p* < 0.05, ^***^ *p* < 0.001. ns, no significant difference.

The cytocompatibility of the piezoelectric hydrogels was evaluated by examining L929 cell viability, proliferation, and adhesion behavior. As shown in Figure 4A, robust growth and firm adhesion of L929 cells were observed on all hydrogel scaffolds in the Live/Dead assay, indicated by strong green fluorescence, which confirms excellent biocompatibility. Further assessment with a CCK‐8 kit revealed a marked increase in L929 cell numbers over the culture duration (Figure 4B). Immunofluorescence staining was used to analyze cell adhesion and morphology. While cells in the pure Gel group displayed a spindle‐like shape (Figure 4C), those on piezoelectric hydrogels adopted a polygonal morphology with visible filopodia, more developed cytoskeletons, and enhanced colony formation, suggesting improved adhesion. Flow cytometry ‐based apoptosis quantification indicated a high viability rate exceeding 95% in all groups (Figure 4D), supporting the conclusion that the hydrogels possess good biocompatibility with minimal cytotoxicity. EdU staining performed after 24 h of co‐culture (Figure 4E) confirmed that localized electrical stimulation significantly boosted cell proliferation. Specifically, cells on piezoelectric hydrogels, especially with 1% CMB Gel, showed higher proliferation rates compared to the Gel group. This effect is attributed to the piezoelectric properties, which facilitate cell cycle progression and enhance the proliferation rate [25]. Together, these findings demonstrate that the designed piezoelectric hydrogels effectively promote L929 cell adhesion and proliferation. Hemocompatibility was also investigated by measuring hemolytic activity. Both visual inspection and quantitative analysis of hemolysis rates revealed results comparable to the PBS negative control (Figure 4F), confirming a safe hemolysis ratio below the 5% threshold [26]. These outcomes verify that the synthesized piezoelectric hydrogel material is non‐hemolytic and exhibits satisfactory compatibility with blood cells.

To confirm the biocompatibility of the hydrogel on the target cell type, we repeated the core assays using primary mouse chondrocytes (isolated from 4 to 6 days old C57BL/6 mice) (Figure S10). As shown in Figure S11, primary chondrocytes were seeded onto the same hydrogels, and cell viability was assessed by Live/Dead staining and CCK‐8 assay. The results were fully consistent with those obtained from L929 cells: the CMB Gel group showed high cell viability (>90%) and no apparent cytotoxicity, while the IL‐1β group exhibited reduced viability. These data confirm that the piezoelectric hydrogel has excellent cytocompatibility toward primary chondrocytes, supporting its translational relevance.

### 2.4 Piezoelectric Hydrogel Reverses the Senescent Phenotype and ECM Degradation in OA Chondrocytes

**FIGURE 5:**
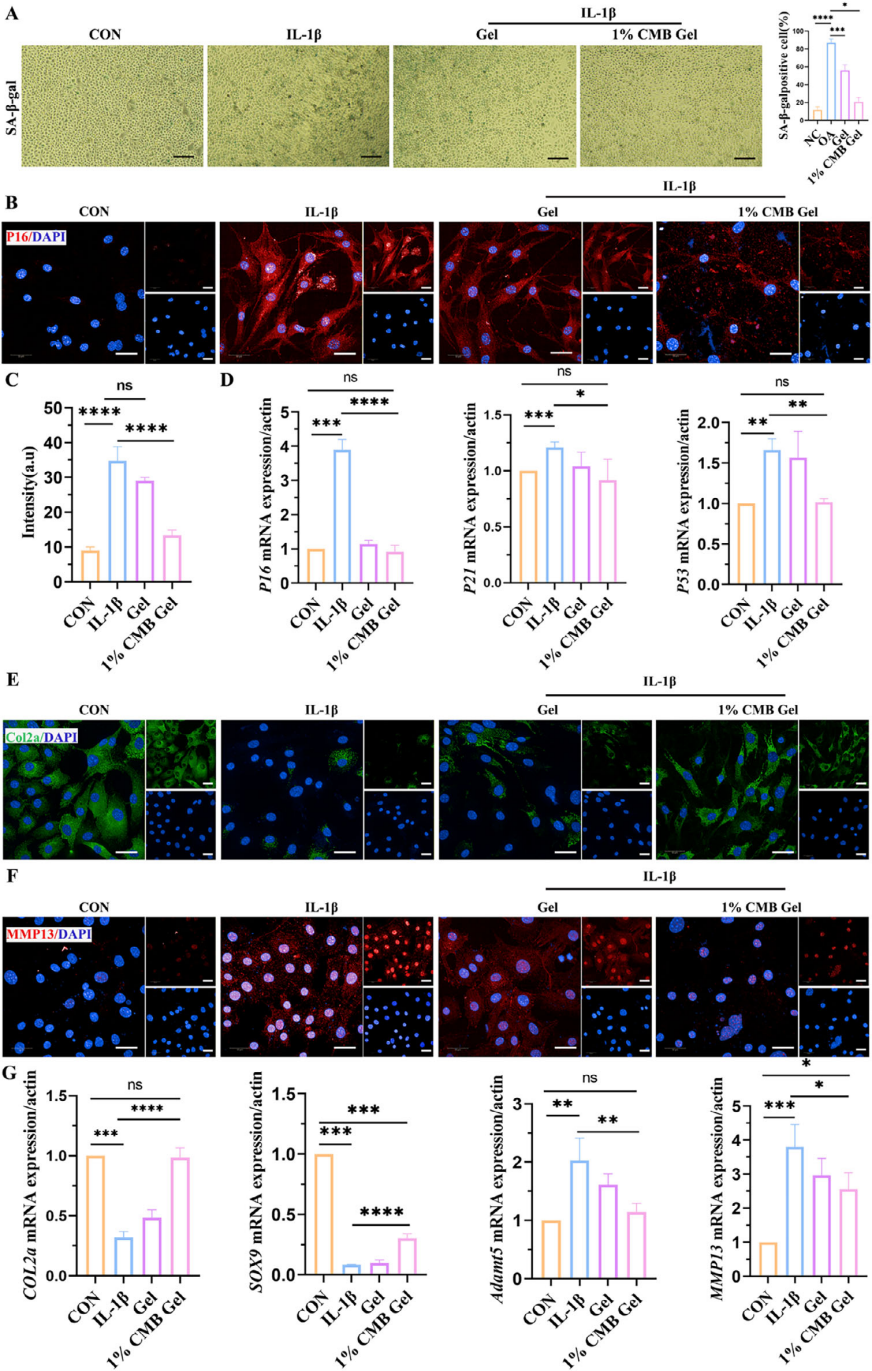
Piezoelectric hydrogel can alleviate the degradation and aging of the extracellular matrix of chondrocytes induced by IL‐1β. (A) Representative image of β‐galactosidase staining of chondrocytes on the hydrogel. Scale bar,200 µm. (B, C) Representative immunofluorescence image showing the markers of chondrocyte senescence P16. Scale bar,50 µm.(D) Detection of the effects of piezoelectric hydrogel on the expression of P16, P21, and P53 in mouse chondrocytes under the stimulation of IL‐1β or not using RT‐qPCR. (E, F) Detection of the effects of piezoelectric hydrogel on the expression of MMP13 (red) and COL2A1 (green) in mouse chondrocytes under the stimulation of IL‐1β or not, using immunofluorescence. Scale bar,50 µm. (G) Detection of the effects of piezoelectric hydrogel on the expression of COL2A1, SOX9, Adamt5, and MMP13 in mouse chondrocytes under the stimulation of IL‐1β or not using RT‐qPCR. Treatment groups are defined as follows: CON, untreated chondrocytes; IL‐1β, cells treated with 30 ng/mL IL‐1β for 24 h; Gel, IL‐1β plus hydrogel without nanoparticles; CMB Gel, IL‐1β plus 1% Cap‐CM@BTO/Gel with ultrasound (1 MHz, 2.5 W/cm^2^, 5 min/session, 3 sessions/day). Data presented as mean ± standard deviation. Statistical analysis was performed using one‐way analysis of variance (ANOVA) combined with Tukey's post hoc test. n = 5, ^*^ *p* < 0.05, ^***^ *p* < 0.001. ns, no significant difference.

In OA, cellular senescence is considered a major factor driving age‐related alterations in cartilage homeostasis, function, and response to injury. Aged chondrocytes in OA typically exhibit poor proliferative activity. Senescent OA chondrocytes demonstrate high expression of SA‐β‐gal. Treatment with the piezoelectric hydrogel reduced IL‐1β induced chondrocyte senescence, significantly decreasing SA‐β‐gal positive cells on day 3 (Figure 5A). p16, a common and reliable senescence marker, is known to promote cellular senescence. Immunofluorescence staining for p16 protein in IL‐1β induced senescent chondrocytes clearly showed reduced p16 protein expression in the piezoelectric hydrogel‐treated group (Figure 5B,C). As a positive marker of cellular senescence, p53 is highly associated with senescent cell cycle arrest, as it accumulates and activate various cyclin‐dependent kinase inhibitors (CDKIs). Polymerase chain reaction (PCR) analysis revealed that the piezoelectric hydrogel downregulated the expression of senescence‐associated genes, including p16, p21, and p53 (Figure 5D). This further demonstrates the role of the piezoelectric hydrogel in mitigating chondrocyte senescence. Analysis of chondrocyte morphology and matrix secretion indicated that the piezoelectric hydrogel suppresses the expression of senescence‐related genes in OA, thereby enhancing OA chondrocyte viability, improving morphological maintenance, promoting cartilage matrix secretion, and upregulating cartilage‐specific gene expression. Chondrocyte hypertrophy and senescence during OA share similar biomarkers and processes [27]. The gene expression of cartilage‐specific markers was investigated: COL2A1 represents type II collagen formation. In the piezoelectric hydrogel group, COL2A1 expression was significantly increased, while MMP13 expression was suppressed (Figure 5E,F). This effect was further validated at the molecular level (Figure 5G). Compared to the control group, the OA model group showed significantly elevated mRNA expression levels of chondrocyte catabolic indicators. The expression levels of MMP13(a zinc metalloproteinase family enzyme involved in extracellular matrix degradation) and ADAMTS5 were also markedly increased. Conversely, the piezoelectric hydrogel significantly downregulated the expression of these genes. To verify that the observed chondroprotective effects are not limited to supraphysiological IL‐1β stimulation, we repeated key experiments using a lower, more pathophysiologically relevant concentration of 5 ng/mL IL‐1β for 24 h. As shown in Figure S12, the 1% CMB Gel plus US treatment significantly reduced MMP13 expression (immunofluorescence) compared to the 5 ng/mL IL‐1β alone group. These results are consistent with those obtained using 30 ng/mL IL‐1β, confirming that our conclusions are robust across different inflammatory intensities.

### 2.5 Piezoelectric Hydrogel Induces Mitophagy

**FIGURE 6:**
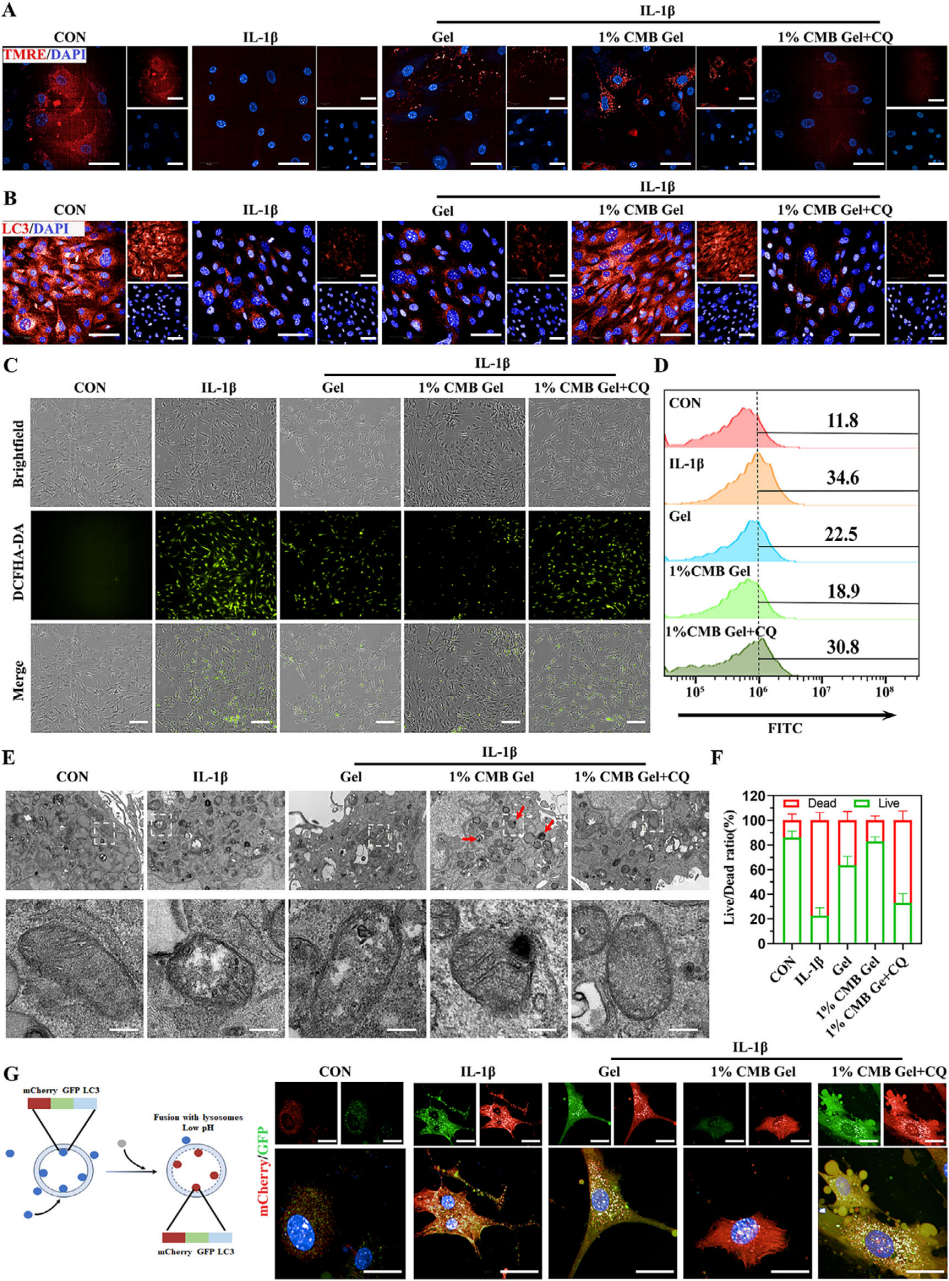
The piezoelectric hydrogel triggers the piezoelectric effect, thereby enhancing mitochondrial autophagy and improving the function of chondrocytes. (A) Representative image of TMRE (red) immunofluorescence staining in chondrocytes. Scale bar, 50 µm. (B) Representative image of LC3 (red) immunofluorescence staining in chondrocytes. Scale bar, 50 µm. (C) Representative image of reactive oxygen species immunofluorescence staining in different groups. Scale bar, 200 µm. (D) Flow cytometric analysis of reactive oxygen levels in different groups. (E) Representative transmission electron microscope images of mitochondria in chondrocytes from different groups. Scale bar, 200 nm. (F) Quantitative data of immunofluorescence staining for the viability/death of chondrocytes in different groups. n = 5. (G) Schematic diagram of the LC3 dual‐labeling system for assessing autophagic flux (left). After transfecting Chondrocytes cells with mCherry‐GFP‐LC3, subsequent experiments included IL‐1β stimulation, ultrasound activation treatment, and assessment of autophagosomes (yellow) and autolysosomes (red) formation. Scale bar,100 µm. Treatment groups: CON, untreated; IL‐1β, 30 ng/mL for 24 h; Gel, IL‐1β + empty hydrogel; CMB Gel, IL‐1β + piezoelectric hydrogel + US (1 MHz, 2.5 W/cm^2^, 5 min, 3×/day); CMB Gel + CQ, same as CMB Gel with chloroquine (10 µm). Data presented as mean ± standard deviation. Statistical analysis was performed using one‐way analysis of variance (ANOVA) combined with Tukey's post hoc test. ^*^ *p* < 0.05, ^***^ *p* < 0.001. ns, no significant difference.

Membrane potential, a bioelectrical signal, plays a crucial role in signal transduction processes involving the sensing and transmission of electrical stimuli [28]. To further validate the effect of the piezoelectric hydrogel on mitochondrial membrane potential, we assessed it using TMRE staining (Figure 6A and Figure S13). In the ultrasound‐assisted piezoelectric hydrogel treatment group, mitochondria in chondrocytes exhibited bright red fluorescence, indicating that the piezoelectric stimulation from the hydrogel caused a hyperpolarizing shift in the mitochondrial membrane potential. The results confirmed that ultrasound‐assisted piezoelectric hydrogel treatment led to the depolarization and subsequent loss of membrane potential in IL‐1β induced damaged mitochondria, thereby promoting mitophagy. Mitophagy is a selective autophagy process responsible for removing damaged mitochondria, maintaining mitochondrial quality through lysosomal degradation and recycling of these organelles [29]. Subsequently, when we performed LC3 staining across different groups, we observed a significant upregulation of LC3 expression specifically in the ultrasound‐assisted piezoelectric hydrogel treatment group (Figure 6B and Figure S14). These results suggest that the treatment promotes mitophagy in chondrocytes under IL‐1β conditions. Previous studies have indicated that damaged mitochondria are a primary source of intracellular reactive oxygen species (ROS), and excessive ROS production disrupts normal cell and tissue function through various mechanisms [30]. Therefore, we used the fluorescent probe 2',7'‐dichlorodihydrofluorescein diacetate (DCFH‐DA) to quantify cellular ROS levels in different groups (Figure 6C,D). The ROS level in the piezoelectric hydrogel group was significantly lower than that in the IL‐1β group. These results demonstrate that ultrasound‐assisted piezoelectric hydrogel treatment effectively suppresses sustained intracellular ROS generation by promoting mitophagy, thereby addressing the issue of excessive ROS production. We examined mitochondria in chondrocytes from different groups using TEM. The results showed that mitochondria in the control group were of normal size with a well‐organized and intact structure. In contrast, mitochondria in the IL‐1β group exhibited swelling, vacuolization, decreased density, and structural disruption (Figure 6E). Notably, in the piezoelectric hydrogel group, damaged mitochondria were transformed into mitochondrial vesicles and sequestered within autophagosomes. This was distinct from the model group, where damaged mitochondria failed to form autophagosomes. Further evidence of activated mitophagy was provided by TFAM immunofluorescence, which displayed intensified and aggregated signals in the hydrogel‐treated cells, indicating the mobilization of this key factor to facilitate mitochondrial turnover (Figure S15). Cell viability was assessed via Live/Dead staining across the groups. The results showed robust cell growth in the control group, whereas the IL‐1β group displayed a large number of apoptotic cells (emitting red fluorescence) (Figure 6F and Figure S16). The number of apoptotic cells was reduced in the piezoelectric hydrogel group. Our confocal laser scanning microscopy (CLSM) results showed that after transfecting chondrocytes with mCherry‐GFP‐LC3, IL‐1β treatment led to the formation of more yellow puncta, representing autophagosomes. The ultrasound‐assisted piezoelectric hydrogel treatment resulted in red puncta, representing autolysosomes, indicating that autophagy had progressed to the stage of autophagosome lysosome fusion (Figure 6G and Figure S17). 3‐MA (an autophagy initiation inhibitor) and chloroquine (CQ), an autophagy inhibitor that primarily blocks autophagosome‐lysosome fusion by raising lysosomal pH and thus inhibits autophagic flux completion [31], abolished the therapeutic effect of the piezoelectric hydrogel when added, confirming its dependence on the autophagy pathway (Figure S18). These results collectively demonstrate that ultrasound‐assisted piezoelectric hydrogel treatment suppresses ROS by promoting mitophagy in chondrocytes under IL‐1β conditions. Importantly, ultrasound alone (without hydrogel) had no significant effect on ROS or MMP13 levels (Figure S19), confirming that the observed benefits require the piezoelectric material.

### 2.6 Piezoelectric Hydrogel Mitigates Osteophyte Formation, Subchondral Bone Remodeling, and Synovitis in DMM Mice

**FIGURE 7:**
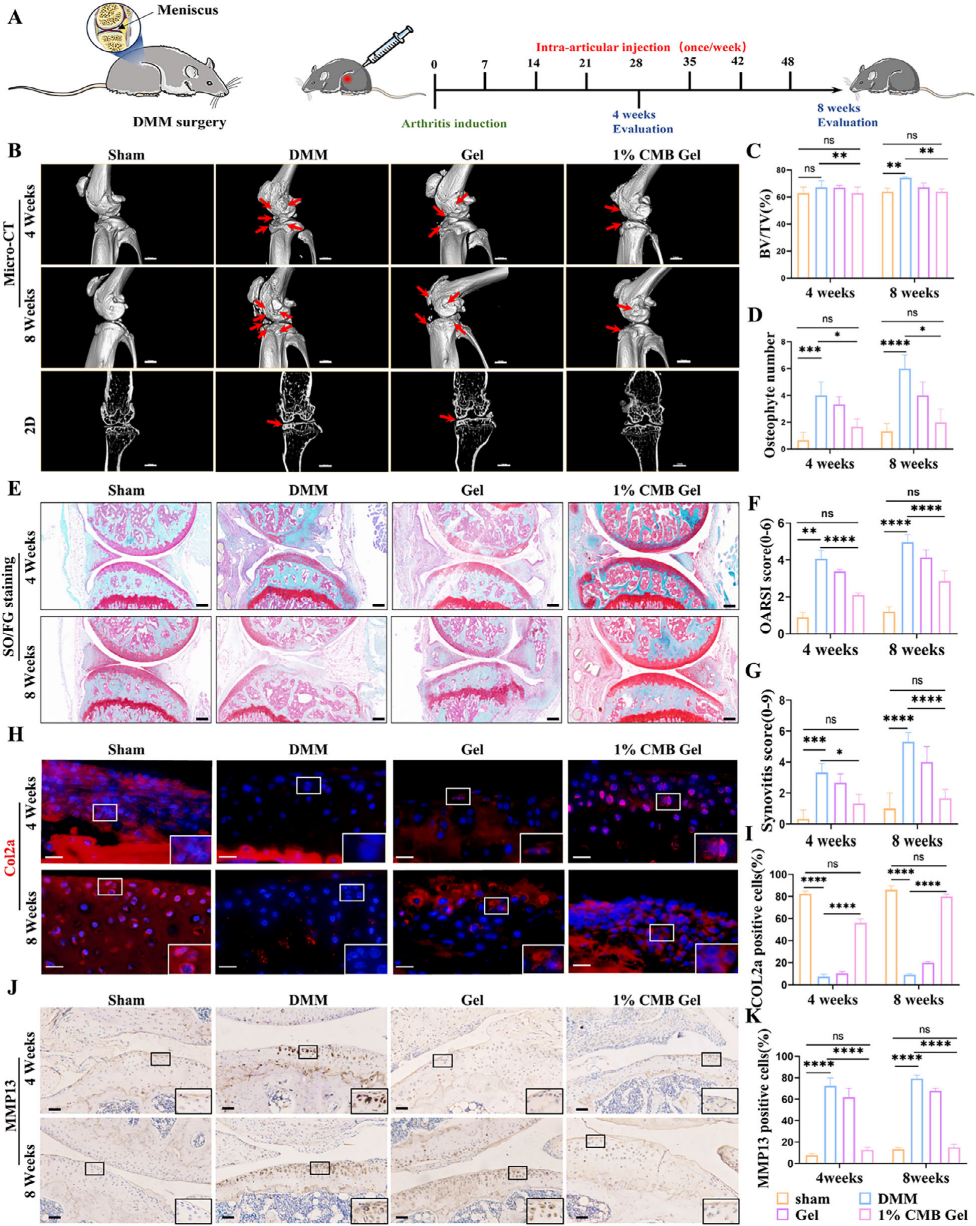
Piezoelectric hydrogel treatment can alleviate the progression of osteoarthritis in mice. (A) Research design for treating osteoarthritis in mice using piezoelectric hydrogel. (B–D) Representative 3D micro‐CT images showing the surface morphology of the joints (n = 6), with the bottom being 2D micro‐CT images. Displaying osteophytes (red arrows) and the microstructure of subchondral bone. Scale bar,1 mm. (E, F) After 4 and 8 weeks of treatment, perform SO&FG staining on the knee joints of each group. Scale bar,200 µm. (H, I) After 4 and 8 weeks of treatment, perform COL2a immunofluorescence staining of the cartilage of the mouse knee joints. Scale bar,50 µm. (J, K) After 4 and 8 weeks of treatment, perform MMP13 immunohistochemical staining of the cartilage of the mouse knee joints. Scale bar, 100 µm. Statistical analysis was performed using one‐way analysis of variance (ANOVA) combined with Tukey's post hoc test. n = 6^*^ *p* < 0.05, ^***^ *p* < 0.001. ns, no significant difference.

An OA mouse model was employed to evaluate the efficacy of the injectable piezoelectric hydrogel. Destabilization of the medial meniscus (DMM) surgery served as a well‐characterized and robust surgical model for inducing OA (Figure 7A and Figure S20). Intra‐articular injections were started at 1 week post DMM and administered once per week thereafter until the endpoint (4 or 8 weeks). As a key component of joint cartilage homeostasis, the subchondral bone provides a firm attachment for cartilage and withstands mechanical stress [32]. During OA pathogenesis, aberrant stress leads to the formation of microcracks in the subchondral bone. With progressive stress, the subchondral bone undergoes remodeling, can develop sclerosis [33]. Micro computed tomography (Micro‐CT) was used to analyze the microarchitecture of the tibial metaphysis. As shown in Figure 7B–D, mice treated with the piezoelectric hydrogel exhibited a markedly superior trabecular microstructure in the tibial metaphysis compared to the OA control group. Hydrogel treatment reduced osteophyte formation in DMM mice, with a more pronounced effect observed in the piezoelectric hydrogel group. Relevant parameters, including trabecular number (Tb.N), trabecular thickness (Tb.Th), and trabecular separation (Tb.Sp), were restored following treatment with the piezoelectric hydrogel (Figure S21). After piezoelectric hydrogel treatment, the area of cartilage degeneration and the total osteophyte width were significantly restored, as indicated by the red areas in Safranin O staining. Histological analysis based on the Osteoarthritis Research Society International (OARSI) scoring system was performed blindly to grade cartilage degeneration (Figure 7E,F). H&E staining of the knee joints at 4 and 8 weeks after treatment further demonstrated improved joint histoarchitecture following piezoelectric hydrogel therapy (Figure S22A). Synovitis was graded by assessing the number of synovial lining cell layers and the degree of synovial tissue hyperplasia. Synovial inflammation, a pathological feature during OA progression, is triggered by cartilage debris stimulation and lymphocytic infiltration in the synovium [34]. The synovitis score demonstrated a decreasing trend after piezoelectric hydrogel treatment. These results indicate that, in addition to rescuing cartilage degeneration, the piezoelectric hydrogel can ameliorate synovial inflammation in OA joints (Figure 7G and Figure S22B).

Additionally, immunohistochemical staining was performed to evaluate cartilage degradation. The number of MMP13 positive cells was significantly higher in the DMM group compared to the sham group, while the number of COL2A1 positive cells was markedly reduced, indicating elevated inflammation and exacerbated cartilage breakdown. In contrast, the piezoelectric hydrogel group showed reduced expression of MMP3 and MMP13, alongside increased expression of COL2A1 and SOX9 (Figure 7H–K and Figure S22D,E). This suggests suppressed inflammation and mitigated cartilage degradation. The joint‐protective effects were further corroborated by p16 immunohistochemical staining of the articular cartilage, which demonstrated that piezoelectric hydrogel treatment effectively reduced the burden of p16‐positive senescent chondrocytes at 4 and 8 weeks after treatment (Figure S22C). To evaluate the long‐term safety of the CMB Gel, we performed histopathological and serum biochemical analyses using samples collected at the 8 weeks endpoint from mice in the sham, DMM, Gel, and 1% CMB Gel groups. Major organs (heart, liver, spleen, lung, and kidney) were sectioned and stained with H&E. As shown in Figure S23A, no obvious pathological abnormalities, inflammatory infiltrates, necrosis, or fibrosis were observed in any of the CMB Gel‐treated mice compared to the sham or DMM groups, indicating good organ compatibility. Blood biochemistry parameters reflecting liver and kidney function, including albumin (ALB), alanine aminotransferase (ALT), aspartate aminotransferase (AST), creatine kinase (CK), globulin (GLB), lactate dehydrogenase (LDH), total protein (TP), and uric acid (UA), were measured. As presented in Figure S23B, all parameters remained within normal ranges and showed no significant differences between the CMB Gel group and the control groups (sham and DMM), confirming no detectable hepatic or renal impairment. Collectively, these data demonstrate that the CMB Gel does not elicit systemic toxicity over the 8 weeks observation period, supporting its biosafety for translational applications, and that the piezoelectric hydrogel presents a promising therapeutic platform for OA, capable of inhibiting cartilage degradation, reducing inflammatory responses, and further improving the microarchitecture of the subchondral bone.

### 2.7 Chondroprotective Effects of Piezoelectric Hydrogels in Clinically Derived OA Cartilage Explants

**FIGURE 8:**
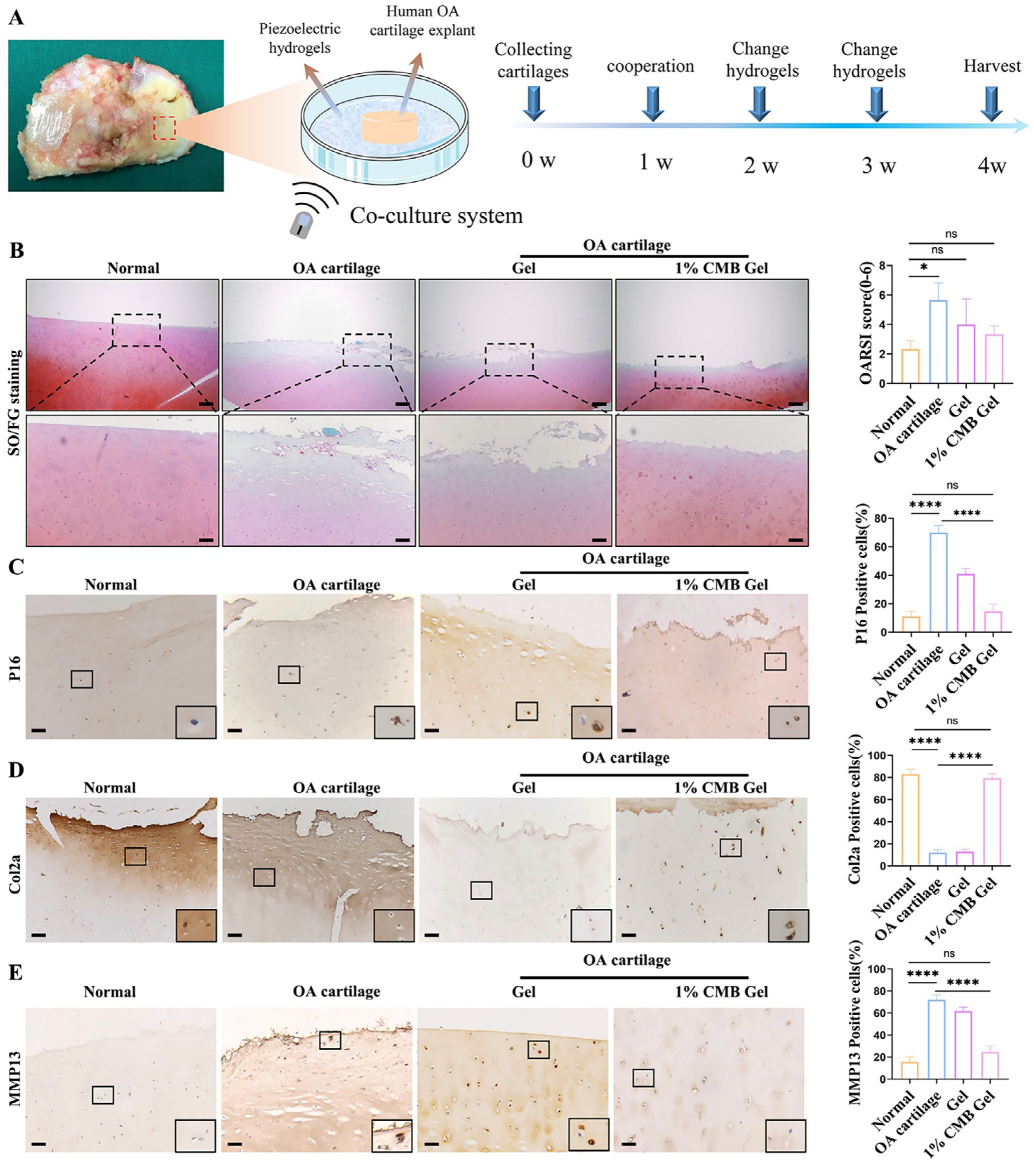
Therapeutic effect of piezoelectric hydrogel in human osteoarthritis cartilage samples. (A) Experimental design schematic. After culturing human osteoarthritis cartilage samples in vitro for one week, the hydrogel was replaced weekly, and cartilage samples were collected after 3 weeks. (B) SO/FG staining of different groups of cartilage samples. Scale bar,200 µm. (C, D, E) Immunohistochemical staining results of P16, COL2A1, and MMP13. Scale bar,100 µm. Statistical analysis was performed using one‐way analysis of variance (ANOVA) combined with Tukey's post hoc test. n = 5, ^*^ *p* < 0.05, ^***^ *p* < 0.001. ns, no significant difference.

Based on the successful therapeutic outcomes in OA mice, we further employed histological and immunohistochemical staining methods, including Hematoxylin and Eosin (H&E) staining, Safranin O/Fast Green staining, and immunohistochemical staining to evaluate the clinical therapeutic potential of the piezoelectric hydrogel. OA‐damaged cartilage explants were collected (Figure 8A). Safranin O/Fast Green staining results indicated varying degrees of improvement in cartilage damage in the hydrogel‐treated groups compared to the OA group (Figure 8B). Specifically, the piezoelectric hydrogel group not only exhibited smooth cartilage surfaces but also showed deeper red staining, similar to the normal group. In H&E staining, significant cartilage defects were observed in the OA group, while fibrotic tissue formation was detected in the hydrogel‐treated groups (Figure S24), suggesting their limited capacity to attenuate matrix degradation. In contrast, cartilage explants treated with the piezoelectric hydrogel formed cartilage‐like tissue with improved surface continuity, demonstrating an enhanced ability to preserve cartilage matrix, likely attributable to the hydrogel's capacity to modulate the local inflammatory response. Of note, the cartilage tissue formed in the piezoelectric hydrogel group was continuous and smooth, with a thickness resembling that of normal articular cartilage. Notably, immunohistochemical staining revealed that the piezoelectric hydrogel group exhibited higher expression levels of COL2A1 (Figure 8D) and SOX9 (Figure S25A). Conversely, the piezoelectric hydrogel reduced the levels of p16 (Figure 8C), MMP13 (Figure 8E), and MMP3 (Figure S25B). These findings are consistent with the therapeutic effects observed for the piezoelectric hydrogel in OA mice and confirm its beneficial effects on matrix preservation.

### 2.8 Mechanistic Insights Into Piezoelectric Hydrogel‐Induced Mitophagy via the mTOR/GATD3A/TFAM Axis

**FIGURE 9:**
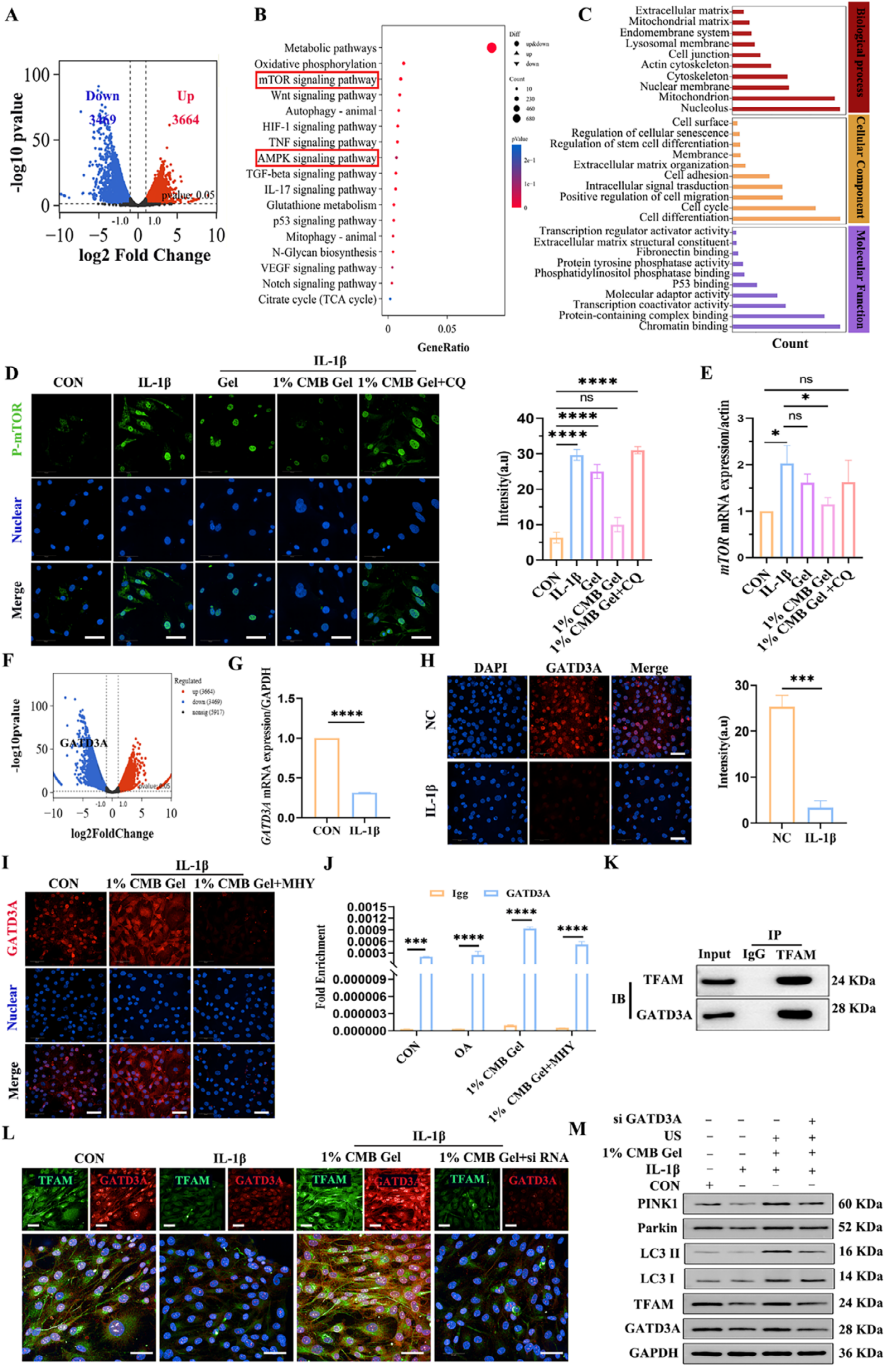
Molecular mechanism of piezoelectric hydrogel promoting cartilage protection in mice. (A) Quantitative analysis of DEGs in chondrocytes of the IL‐1β group and the piezoelectric hydrogel group. (B) Analysis of KEGG pathways in chondrocytes of the IL‐1β group and the piezoelectric hydrogel group. (C) Top 30 upregulation gene ontology terms. (D) Immunofluorescence image of P‐mTOR (green). Scale bar, 50 µm. (E) RT‐qPCR analysis of P‐mTOR levels in chondrocytes. (F) Volcano plot of RNA‐Seq data. (G) RT‐qPCR analysis of GATD3A expression in mouse chondrocyte samples stimulated by IL‐1β (for 24 h). (H) Immunofluorescence staining of GATD3A expression in mouse chondrocyte samples stimulated by IL‐1β (for 24 h). Scale bar, 50 µm. (I) Immunofluorescence staining of GATD3A and GATD3A level in chondrocytes using ultrasound combined with piezoelectric hydrogel and MHY1485 (mTOR activator) (with or without IL‐1b stimulation). Scale bar, 50 µm. (J) ChIP‐qPCR analysis showing the enrichment level of H3K27ac in the GATD3A promoter region of chondrocytes under different treatment conditions. (K) Co‐IP of GATD3A and TFAM in chondrocyte lysates using anti‐GATD3A antibody or normal IgG (negative control). Immunoblotting analysis using TFAM and GAPDH antibodies. (L) Representative immunofluorescence image showing the subcellular localization of GATD3A (red) and TFAM (green) in chondrocytes. Scale bar, 50 µm. (M) Immunoblot analysis of LC3‐I/II, PINK1/Parkin, TFAM, and GATD3A protein levels in chondrocytes under different treatment conditions. Statistical analysis was performed using one‐way analysis of variance (ANOVA) combined with Tukey's post hoc test. n = 3, ^*^ *p* < 0.05, ^***^ *p* < 0.001. ns, no significant difference.

Following the investigation into the effect of the piezoelectric hydrogel on chondrocyte mitophagy, we further analyzed and sought to deepen the understanding of its underlying mechanism. RNA‐Seq data were utilized to explore the potential mechanisms of mitochondrial dysfunction in osteoarthritic chondrocytes. Transcriptomic analysis based on RNA‐Seq revealed that piezoelectric hydrogel treatment induced significant transcriptome reprogramming in OA chondrocytes, with a total of 3664 genes upregulated and 3469 genes downregulated (Figure 9A). To investigate the potential mechanisms, clustering analysis was performed on the differentially expressed genes (DEGs). KEGG enrichment analysis and heatmaps indicated that under piezoelectric influence, key regulatory pathways were enriched, including the mTOR signaling pathway and the AMPK signaling pathway (Figure 9B). In the gene ontology analysis, the significantly upregulated gene ontology terms include “mitochondrion”, “extracellular matrix”, and “extracellular matrix structural components”. These findings highlight the crucial role of piezoelectric hydrogels in promoting the formation of the extracellular matrix and maintaining mitochondrial homeostasis, as the formation of the extracellular matrix and mitochondrial homeostasis are important aspects of cartilage tissue development (Figure 9C).

KEGG pathway analysis revealed a significant interaction between the AMPK and mTOR signaling pathways. Given that AMPK is a known upstream negative regulator of mTOR, we hypothesized that the observed pathway changes might be coordinated by a common upstream signal. Since the piezoelectric hydrogel generates a local electric field upon ultrasound activation, and voltage‐gated calcium channels are highly sensitive to electric fields, we speculated that the electrical signal may trigger Ca^2^^+^ influx, leading to AMPK activation and subsequent mTOR inhibition. To test this hypothesis, we measured intracellular Ca^2^^+^ using Fluo‐4 AM. Compared to the IL‐1β or control groups, CMB Gel + US treatment induced a rapid and significant increase in Ca^2^^+^ concentration (Figure S26A). Western blot analysis showed that CMB Gel + US upregulated p‐AMPK and downregulated p‐mTOR, and both effects were fully reversed by the Ca^2^^+^ chelator BAPTA‐AM (Figure S26B). Collectively, these data support a model in which ultrasound‐activated piezoelectric potentials open calcium channels; the resulting Ca^2^^+^ influx activates AMPK, which in turn inhibits mTOR. Since Phosphorylation of mTOR at Ser2448 is associated with its kinase activity, we detected P‐mTOR expression in chondrocytes by immunofluorescence. Staining showed that the fluorescence intensity of P‐mTOR in chondrocytes decreased after treatment with the piezoelectric hydrogel plus ultrasound compared to the OA model group (Figure 9D). This result was validated at the animal level: immunofluorescence staining of mouse knee joint tissues showed a significant downregulation in the rate of P‐mTOR positive cells in the cartilage layer after intra‐articular injection of the hydrogel (Figure S27). Consistent results were obtained at the molecular level (Figure 9E), indicating that the localized electric field generated by the piezoelectric hydrogel under ultrasound assistance effectively inhibits the mTOR signaling pathway.

Volcano plots of DEG analysis showed that GATD3A expression was significantly reduced (Figure 9F). Previous studies suggest that GATD3A may play a potential role in regulating mitochondrial function [35]. qPCR analysis of chondrocytes confirmed decreased GATD3A expression in the IL‐1β induced OA model group (Figure 9G). These changes were further corroborated by immunofluorescence and immunohistochemical detection of GATD3A protein expression (Figure 9H and Figure S28A,B). Given the crucial role of GATD3A in regulating mitochondrial function, we investigated its role in mitophagy and the potential mechanism. After intervention with the piezoelectric hydrogel plus ultrasound, GATD3A protein levels recovered to near‐normal levels. This upregulation could be completely blocked by the mTOR activator MHY1485 (MHY), indicating its dependence on mTOR inhibition (Figure 9I and Figure S29). Therefore, we hypothesize that the piezoelectric effect of this hydrogel will generate an electrical signal under the action of ultrasound, thereby inhibiting the mTOR pathway. Subsequently, the inhibitory effect of mTOR will relieves the suppression of GATD3A, leading to an increase in the expression of GATD3A. This upregulation of GATD3A plays a central role. To verify this hypothesis, we conducted ChIP‐qPCR analysis (Figure 9J). The results revealed that H3K27ac enrichment at the GATD3A promoter was significantly lower in the OA model group, consistent with the decreased GATD3A mRNA expression. After intervention with the piezoelectric hydrogel plus ultrasound, H3K27ac enrichment levels increased substantially, suggesting that piezoelectric stimulation remodels the epigenetic landscape of the GATD3A promoter to an open state. Notably, the mTOR activator MHY1485 completely reversed the hydrogel‐induced H3K27ac enrichment, confirming that this process depends on mTOR signaling inhibition. A positive correlation between GATD3A and LC3 mRNA levels was observed in the GEPIA database (R = 0.89), suggesting a potential association that warrants further experimental investigation (Figure S28C). Mitochondrial transcription factor A (TFAM) binds to the autophagy protein LC3 via its LC3‐interacting region (LIR) motif, thereby mediating the autophagic clearance of mtDNA [36]. We speculated that the GATD3A/TFAM axis might play a key role in driving chondrocyte mitophagy. To validate the regulatory relationship between GATD3A and TFAM, we conducted Co‐IP experiments. As shown in Figure 9K, TFAM was specifically co‐precipitated from chondrocyte lysates using an anti GATD3A antibody (experimental group), whereas no TFAM signal was detected when using normal IgG for precipitation, confirming the specificity of the interaction. Furthermore, the unrelated protein GAPDH was not detected in the same precipitates, further ruling out non‐specific binding. Additionally, triple immunofluorescence staining of chondrocytes for GATD3A (red), TFAM (green), and DAPI (nucleus, blue) demonstrated that hydrogel treatment significantly enhanced the co‐localization of GATD3A and TFAM (Figure 9L and Figure S30). This indicates that GATD3A and TFAM form a complex within cells, which may provide the structural basis for GATD3A‐mediated stabilization of TFAM and the promotion of mitophagy, ultimately improving the chondrocyte microenvironment and delaying OA progression. The PINK1/Parkin pathway plays a critical role in initiating mitophagy. Upon mitochondrial damage, PINK1 accumulates on the mitochondrial surface and promotes the recruitment of Parkin, which ubiquitinates mitochondrial outer membrane proteins, subsequently triggering mitophagy. Based on this, we examined whether GATD3A influences the PINK1/Parkin pathway. As expected, GATD3A upregulation significantly enhanced PINK1/Parkin‐mediated mitophagic flux: Western Blot showed an increased LC3‐II/LC3‐I ratio (Figure 9M and Figure S31). These effects were attenuated in GATD3A‐knockdown cells (Figure S32), confirming the central role of GATD3A.In summary, this study elucidates a novel mechanistic pathway: ultrasound‐activated piezoelectric hydrogel generates a local electric field, triggering the opening of voltage‐gated calcium channels on the chondrocyte membrane and inducing Ca^2^^+^ influx. Ca^2^^+^ then activates AMPK through the CaMKKβ pathway, and activated AMPK subsequently inhibits the mTOR signaling pathway. mTOR inhibition leads to increased H3K27 acetylation in the GATD3A promoter region, epigenetically upregulating GATD3A expression. Acting as a central molecular bridge, GATD3A coordinately promotes TFAM mediated mitochondrial DNA maintenance and PINK1/Parkin‐mediated mitophagy, ultimately clearing damaged mitochondria and restoring chondrocyte function. This work thereby provides an innovative OA therapeutic strategy that synergistically combines mechano‐responsiveness (ultrasound‐activated piezoelectricity) with precise molecular intervention (the Ca^2^^+^/AMPK/mTOR/epigenetics/mitophagy axis).

## 3 Conclusion

In summary, we have successfully developed an injectable, piezoelectric hydrogel (Cap‐M@BTO/Gel) that integrates active cartilage targeting with ultrasound‐responsive piezoelectric therapy for the treatment of osteoarthritis. The hydrogel is constructed from oxidized chondroitin sulfate and collagen via dynamic borate ester bonds, enabling excellent injectability and biocompatibility. The piezoelectric component, composed of chondrocyte membrane‐coated barium titanate nanoparticles functionalized with CAP‐targeting peptides, significantly enhances accumulation and retention within damaged cartilage.

Mechanistically, we demonstrated that the piezoelectric signals generated under ultrasound stimulation induce Ca^2^^+^ influx via voltage‐gated calcium channels, leading to AMPK activation. Activated AMPK subsequently inhibits the mTOR signaling pathway, which results in epigenetic reprogramming of the GATD3A promoter via increased H3K27 acetylation. This epigenetic activation upregulates GATD3A expression, which in turn stabilizes TFAM and enhances PINK1/Parkin‐mediated mitophagy. This cascade effectively clears damaged mitochondria, alleviates oxidative stress, and restores chondrocyte function.

In both IL‐1β induced chondrocyte models and surgically induced Mouse OA models, the Cap‐M@BTO/Gel system significantly mitigated cartilage degradation, reduced osteophyte formation, suppressed synovitis, and promoted cartilage matrix preservation. Moreover, the therapeutic efficacy was validated in human OA cartilage explants, highlighting its clinical translational potential.

This study reveals a novel axis connecting piezoelectric stimulation, epigenetic reprogramming, and mitophagy, and provides a multifunctional biomaterial platform that integrates mechanical responsiveness with molecular precision for treating osteoarthritis. These findings not only advance the understanding of OA pathogenesis but also offer a promising strategy for targeted, non‐invasive regenerative therapy.

In the context of existing literature, our work addresses several critical gaps. Piezoelectric materials such as BTO have been previously applied to bone and cartilage regeneration [37], but the underlying subcellular mechanisms, particularly those regarding mitochondrial quality control, remained largely unexplored. Our study demonstrates that ultrasound‐activated BTO nanoparticles directly inhibit mTOR, a key negative regulator of autophagy, thereby linking piezoelectric stimulation to mitophagy. This finding is consistent with reports that electrical signals can activate AMPK [38], which we confirmed using the Ca^2^^+^ chelator BAPTA‐AM (Figure S26). Moreover, the epigenetic reprogramming of GATD3A via H3K27 acetylation represents a previously unknown mechano‐epigenetic mechanism. Our co‐IP confirmed the association of GATD3A with TFAM stability, suggesting that piezoelectric stimulation can enhance mitochondrial biogenesis through epigenetic upregulation of a nuclear‐encoded mitochondrial protein. The chondrocyte membrane coating strategy, inspired by cancer cell membrane camouflage [13], overcomes the dense ECM barrier and prolongs intra‐articular retention. This addresses a major challenge in OA drug delivery. Several limitations should be noted. The 30 ng/mL IL‐1β concentration is supraphysiological, but key findings were validated with 5 ng/mL IL‐1β (Figure S12). Long‐term biosafety of BTO nanoparticles requires further evaluation in large animals; our preliminary data (Figure S23) showed gradual degradation and minimal systemic accumulation, but longer‐term studies are needed. Future work should also include female and aged animals to address sex and age‐related differences. Nonetheless, this study establishes a new mechano‐electrical‐epigenetic axis for OA therapy and provides a versatile platform for non‐invasive, targeted regenerative medicine.

## 4 Experimental Section

### 4.1 Materials

Barium titanate nanoparticles were purchased from Sigma‐Aldrich, St. Louis, USA (9012‐76‐4).CAP‐sulfydryl (CAP‐SH, DWRVIIPPRPSA‐C) was purchased from Nanjing Yuan peptide Biotech Co. Ltd (Nanjing, China). Fetal bovine serum, α‐modified Eagle's medium (α‐MEM), Dulbecco's modified Eagle's medium (DMEM), phosphate‐buffered saline (PBS), trypsin‐ethylenediaminetetraacetic acid (EDTA), and penicillin/streptomycin (P/S) used for cell culture experiments were all purchased from Gibco Life Technologies, Grand Island, USA (10100147, 12571063, 11965092, 70011044, 17892, 15070063). The CCK‐8 cell proliferation and cytotoxicity detection kit (CA1021) and DAPI solution (C0065) were purchased from Beijing Solarbio Biotechnology Co. Ltd(Beijing, China). Cell senescence detection kit (β‐galactosidase method) (KTA3030), Edu cell proliferation imaging analysis kit (KTA2030), live/dead cell double staining kit (KTA1001), and Annexin V‐AbFluorTM488/PI double staining apoptosis detection kit (KTA0002) were purchased from Abbkine Biotechnology Co. Ltd. (Wuhan, China). The Alexa Fluor 488‐labeled goat anti‐mouse IgG (H+L) (A0428) and the antibody diluent were purchased from Shanghai Biyuntian Biotechnology Co. Ltd(Shanghai, China). Rhodamine phalloidin (T10446) was obtained from Pusitong Biotechnology Co. Ltd. (Beijing, China). The anti‐COL2A1 antibody (AB34712) was purchased from Abcam (USA). The anti‐P16 antibody (YM8152) was obtained from ImmunoWay Biotechnology Company (USA). The anti‐MMP13 antibody (18165‐1‐AP) and the anti‐P‐mTOR (Ser2448) antibody (67778‐1‐Ig) were purchased from Wuhan Proteintech Biotechnology Co. Ltd. (Wuhan, China). The anti‐GATD3A antibody (bsm‐15124R) and the anti‐LC3B antibody (bsm‐6084R) were purchased from Beijing Bioss Biotechnology Co. Ltd. (Beijing, China). The anti‐TFAM antibody (sc‐166965) was purchased from Santa Cruz Biotechnology (USA). The anti‐SOX9 antibody (GB11280‐100) and the anti‐MMP3 antibody were purchased from Wuhan Servicebio Biotechnology Co. Ltd. (Wuhan, China). All the water used in the experiments was purified through the Milli‐Q circulating purification system (Millipore, USA).

### 4.2 Isolation and Purification of Chondrocytes

All animal experiments were approved by the Animal Ethics Committee of Hunan University. Neonatal C57BL/6 mice (4–6 days old) were used for chondrocyte isolation. Briefly, the tibial plateaus were dissected from the mice, rinsed with phosphate‐buffered saline (PBS), and digested with 0.25% trypsin‐EDTA (Gibco, USA) in Eagle‘s medium for 30 min at 37°C. After centrifugation, the trypsin was discarded, and the collagenase II (from Thermo Fisher Scientific in Waltham, Massachusetts, USA) was used to digest for 12 h at 37°C. After digestion, the precipitate was collected by centrifugation and resuspended in complete medium. Then, the cells were transferred to 60 mm culture dishes and uniformly mixed in DMEM containing 10% FBS, 100 U/mL penicillin, and 100 U/ml streptomycin. The cells were placed in a 37°C, 5% CO_2_ cell culture box for cultivation, with the medium changed every other day. The primary chondrocytes were treated with 30 ng/mL IL‐1β (from R&D Systems) for 24 h to create an in vitro osteoarthritis chondrocyte model [39].

### 4.3 Plasma Membrane Protein Isolation

The plasma membrane fraction was isolated using the Minute Plasma Membrane Protein Isolation Kit (Invent Biotechnologies, Eden Prairie, MN) according to the manufacturer's instruction. All steps were performed at 4°C. Briefly, cultured cells were lysed in buffer A and placed in a filter cartridge. After centrifugation at 14 000 rpm for 30 s, pellets were resuspended and centrifuged at 3000 rpm for 1 min. The supernatant was collected and centrifuged again at 14 000 rpm for 10 min. The supernatant was then collected as the cytosol protein fraction and the pellet as the total membrane fraction, which was resuspended in buffer B and centrifuged at 10 000 rpm for 5 min. The resultant pellet was collected separately as an organelle membrane protein for further analysis. The supernatant was then centrifuged again at 14 000 rpm for 15 min, and the final pellet was collected as plasma membrane (PM) protein fraction for subsequent experiments.

### 4.4 The Coating of Nanoparticles With Membranes

Prepare the membrane solution: dilute the membrane protein stock to a concentration of 0.4 mg per 20 µL per sample. Mix 200 uL of a 5 mg/mL BTO NPs suspension with the membrane solution. Use a 400 nm pore size extruder (AVANTI liposome extruder from the United States) to extrude the mixture through 20 cycles to obtain membrane‐coated nanoparticles. Centrifuge the samples at 11 000 g for 15 min to remove excess membrane. After removing the supernatant, collect and store the chondrocyte membrane‐coated BTO NPs in PBS for subsequent studies.

### 4.5 Synthesis of Chondrocyte‐Targeting Polymer

Cholesterol PEG‐maleimide (CLS‐PEG‐MAL) was dissolved in PBS, and CAP‐SH was dissolved in 5 mm Tris(2‐carboxyethyl) phosphine (TCEP). The two were mixed in a 1:1 ratio of substance and shaken at room temperature for 10 h. The solution was then transferred into a dialysis bag (Mw = 3 kDa) to dialyze in PBS for 24 h, before lyophilizing to obtain powdery solid cholesterol‐PEG‐CAP (CLS‐PEG‐CAP).

### 4.6 Surface Modification of CM@NPs

Mix CM@NPs with CLS‐PEG‐CAP at a protein content ratio of 10:1, and incubate at 4°C overnight to achieve surface modification of the nanoparticles through hydrophobic interaction. The particle size and surface potential of the nanoparticles (CAP‐CM@NPs) before and after modification were measured using a Malvern particle size analyzer (Malvern Zetasizer Nano ZS90, UK).

### 4.7 Cell Targeting and Internalization

To evaluate the targeting and internalization efficiency of different types of nanoparticles on chondrocytes, primary chondrocytes from mice were co‐incubated with Cy5.5‐labeled nanoparticles at 37°C. The BTO core was labeled with Cy5.5 (red), and the chondrocyte membrane (CM) was labeled with PKH67 (green). After incubation, the cells were washed three times with PBS, fixed, and stained with DAPI (blue) for nuclear visualization. Confocal fluorescence microscopy was then performed. For flow cytometry analysis, the same treated cells were centrifuged at 1000 rpm for 3 min, resuspended in fluorescence‐activated cell sorting (FACS) buffer, and then analyzed on a flow cytometer (BD Biosciences).

### 4.8 Scanning Electron Microscope

To observe the ultrastructural distribution of different nanoparticles on the cartilage surface, cartilage explants were obtained from the femoral heads of 8 weeks old male C57BL/6 mice and, for human samples, from the femoral condyles of osteoarthritis patients undergoing total knee replacement surgery (informed consent was obtained). The explants were placed in DMEM with 10% fetal bovine serum (FBS) and 1% antibiotics (penicillin‐streptomycin, 10 000 U/mL) in 48‐well plates and cultured at 37°C in 5% CO_2_. Cartilage tissues were then treated with different nanoparticles (200 µg/mL) for 12 h. After treatment, the explants were rinsed three times with phosphate‐buffered saline (PBS) (pH 7.4), immediately fixed with 2.5% glutaraldehyde for 12 h, immersed in sucrose solution, dehydrated through a graded ethanol series, and freeze‐dried. The dried samples were then coated with gold for 60 s using a sputter coater. Finally, the surface morphology and nanoparticle distribution were examined using a scanning electron microscope (SEM, Hitachi, Japan).

### 4.9 Knee Joint Retention Assay

For the purpose of conducting long‐term in vivo retention tests and biodistribution experiments, 5 mg/mL of Cy5.5 labeled nanoparticles were injected into the knee joints of mice. The radiant efficiency within each joint was continuously detected and quantified using Maestro IVIS imaging system (CRI, Woburn, MA, USA) and Living Image software for a period of 5 days. To track the in vivo retention of the hydrogel, Cy5.5 NHS ester was covalently coupled to the amino groups on the hydrogel scaffold (collagen or chondroitin sulfate). The radiant efficiency was similarly monitored and quantified using the Maestro IVIS system over a period of 7 days.

### 4.10 Preparation of APBA Grafted Oxidized CS (APBA‐OCS)

APBA‐OCS was prepared by improving the method of Lee et al. [40]. EDC (0.085 g) and NHS (0.05 g) were added to the OCS aqueous solution (4 mg/mL, 50 mL). After the pH of the mixture was adjusted to 4–5, the mixture was stirred at room temperature (RT) for 1 h in the dark. Then, APBA solution (in DMSO, 0.012 g/mL, 5 mL) was added into the above mixture. The pH was adjusted to 4–5 again, and the reaction continued overnight at RT in the dark. Finally, the reaction solution was dialyzed (MWCO 3500) against distilled water and freeze‐dried to obtain orange powder of APBA‐OCS.

### 4.11 Extraction and Characterization of Acid‐Soluble Collagen From Tilapia Fish Skin

Collagen was extracted from tilapia fish skin according to our previously reported method [41]. Fresh tilapia were descaled, and the skin was washed with DI water and stirred in 0.1 m NaOH for 6 h. After washing, the skin was soaked in butanol/isopropanol for 1 day to remove fats, then transferred to 0.5 m acetic acid (400× volume) and stirred overnight until collagen dissolved. The crude collagen solution was centrifuged, and the supernatant was precipitated with 1 m NaCl. Precipitated collagen was redissolved in 0.5 m acetic acid, dialyzed against 0.1 m acetic acid for 24 h and then against distilled water for 48 h, and lyophilized to obtain pure collagen.

### 4.12 Preparation of Piezoelectric Hydrogel Scaffolds

First, collagen was dissolved in 0.02 m acetic acid solution at 4°C to obtain a 5 mg/mL acidic collagen solution. Then, the acidic collagen solution was mixed with 10× PBS solution at a ratio of 8:1 (v/v) at 4°C. The pH was adjusted to neutral using 1 N NaOH solution, resulting in a collagen solution (Col). Next, the collagen solution was mixed with APBA‐OCS solution. Cap‐CM@BTO was added to the hydrogel solution at final concentrations of 0.1% w/v, 0.5% w/v, and 1% w/v, respectively. After stirring for 10 min to ensure complete mixing, the solution was poured into molds to form hydrogels. In the subsequent part, the collagen‐combined hydrogel with APBA‐OCS is referred to as Gel, while the hydrogels with 0.1% w/v, 0.5% w/v, and 1% w/v cap‐CM@BTO added are respectively called 0.1%cap‐M@BTO/Gel, 0.5%cap‐M@BTO/Gel, and 1%cap‐M@BTO/Gel.

### 4.13 Structure and Morphology of the Hydrogel Scaffolds

The formation of the different hydrogels was confirmed by FTIR. To prepare samples for morphology observation, the hydrogel scaffolds were quenched by immersion in liquid nitrogen for 5 min and then lyophilized for 24 h. Scanning electron microscopy (SEM, MIRA4 LMH, TESCAN, Czech) combined with energy dispersive X‐ray spectroscopy (EDS, Ultim Max 40, UK) were used to analyze the cross‐sectional morphology and nanoparticle distribution in hydrogel scaffolds.

### 4.14 Piezoelectric Properties of Hydrogel Scaffolds

Output voltage was measured with a high‐precision multimeter (DMM7510, Keithley, USA) on cylindrical samples (3 mm thick, 10 mm diameter) with copper foil electrodes. The piezoelectric coefficient (d_33_) was determined by a piezoelectric tester (ZJ‐3AN, JKZC, Beijing, China).

### 4.15 Cell Culture

Primary mouse chondrocytes were isolated from the femoral heads and tibial plateaus of 4–6 days old male C57BL/6 mice. For the establishment of an in vitro osteoarthritis (OA) model, primary chondrocytes were treated with 30 ng/mL recombinant mouse IL‐1β (R&D Systems, USA) for 24 h. Throughout this study, chondrocytes were divided into four treatment groups: the CON group consisted of untreated chondrocytes (no IL‐1β, no hydrogel); the IL‐1β group was stimulated with 30 ng/mL IL‐1β for 24 h to establish an inflammatory model; the Gel group received the same IL‐1β stimulation plus the hydrogel without nanoparticles; and the CMB Gel group was treated with IL‐1β plus 1% Cap‐CM@BTO/Gel followed by ultrasound stimulation (1 MHz, 2.5 W/cm^2^, 5 min per session, 3 sessions per day). In addition, the CMB Gel + CQ group received the same treatment as the CMB Gel group, with the addition of chloroquine (CQ, 10 µm) added before ultrasound stimulation. All experiments were performed in triplicate. All cell cultures were routinely tested for mycoplasma contamination and were negative.

### 4.16 Biocompatibility Study

Following ISO 10993 and GB/T 16886.5, L929 cells were used for biocompatibility evaluation. Cells were seeded onto hydrogels at a density of 3 × 10^3^ cells per scaffold and cultured for 1, 3, and 5 days. CCK‐8 assays were performed, and the absorbance at 450 nm was recorded. Live/Dead staining was carried out on days 1, 3, and 5, and images were captured using a confocal laser scanning microscope (CLSM). Apoptosis was detected by Annexin V/PI staining followed by flow cytometry. EdU staining was performed using the Abbkine KTA2030 kit. To confirm biocompatibility on the target cell type, primary mouse chondrocytes were seeded onto the same hydrogels at a density of 1 × 10^4^ cells per scaffold and assessed similarly by CCK‐8 and Live/Dead staining. All experiments were performed in triplicate.

### 4.17 Cell Adhesion

L929 cells were seeded onto different hydrogels at a density of 1 × 104 cells per scaffold. After 3 days of culture, nuclear and F‐actin staining were performed. The procedure was as follows: First, the cells on the scaffolds were fixed with 4% paraformaldehyde solution (Solarbio, China) for 30 min. Then, they were washed with PBS, permeabilized with 0.1% Triton X‐100 for 15 min, and blocked with 1% BSA solution for 1 h. FITC‐labeled phalloidin (Sigma–Aldrich, USA) was added for F‐actin staining at room temperature for 1 h, followed by DAPI staining for the cell nuclei. The morphology of the cytoskeleton was observed using CLSM.

### 4.18 Ex Vivo Hemolysis Rate

The blood compatibility was studied through an acute hemolysis experiment. In brief, 1 mL of fresh anticoagulated whole blood (collected from mice) was suspended in 10 mL of physiological saline to obtain a homogeneous suspension. The prepared hydrogels were incubated with the aforementioned diluted blood suspension at 37°C for 1 h, then the sample was taken out and centrifuged at 3000 rpm for 10 min. The absorbance of the supernatant was measured at 540 nm, and the hemolysis ratio was calculated using the following formula: Ex vivo hemolysis ratio (%) = (H1 – H0) / (H2 – H0) × 100%, where H0, H1, and H2 represent the absorbance values of the sample, negative control (PBS), and positive control (deionized water), respectively.

### 4.19 Calcium Imaging

Intracellular Ca^2^^+^ was detected with Fluo‐4 AM (2 µm, 30 min, 37°C). After washing, cells were imaged by confocal microscopy. For Ca^2^^+^ chelation, cells were pre‐treated with 20 µm BAPTA‐AM for 1 h before ultrasound [42].

### 4.20 Senescence‐Associated β‐galactosidase (SA‐βGal) Staining

SA‐βGal activity was measured using a staining kit (Solarbio). Cells were plated and allowed to adhere for 24 h before any treatment. IL‐1β (30 ng/mL) was then added for 24 h to induce senescence. After IL‐1β removal, cells were further cultured for an additional 72 h, and staining was performed on day 3 (96 h after initial IL‐1β addition). For staining, cells were fixed for 10 min at room temperature, washed, and then incubated with staining solution overnight at 37°C.

### 4.21 Immunohistochemical and Immunofluorescence (IF) Analyses

The knee joint tissues were fixed in 4% paraformaldehyde for 48 h, decalcified for 21 days, dehydrated, and embedded in paraffin. Sections (3 µm thick) were prepared, stained with safranin O/fast green, hematoxylin, and eosin (H&E) for morphological analysis. The sections were washed three times with PBS for 5 min each, then subjected to antigen retrieval in a 60°C citrate water bath overnight. After three PBS washes, the slides were treated with 3% hydrogen peroxide at room temperature for 10 min, washed three times with PBS, and then incubated at room temperature for 1 h with the primary antibody. The primary antibody or the fluorescent secondary antibody for IHC was applied at room temperature for 1 h, and then the IHC‐stained slides were stained with DAB and hematoxylin, and the IF slides were treated with 4,6‐diamidino‐2‐phenylindole (DAPI, Thermo Fisher Scientific, Waltham, MA, USA) staining solution and sealed (Solarbio, Beijing, China). Quantitative histological analysis of the tissue was performed blindly using the OsteoMeasureXP software (OsteoMeasure, Inc., Atlanta, USA).

### 4.22 RNA‐Mediated Interference

RNA interference experiments were conducted according to a previously described method [43]. When the chondrocyte confluence reached 50%, Lipofectamine RNAiMAX (Thermo Fisher Scientific, catalog number 13778075) was used to transfect the cells with the siRNA oligonucleotides. The siRNA sequences (provided by GenePharma) are listed in Table S2.Stealth siRNA negative control (siNC) duplexes with a similar GC content were used as controls.

### 4.23 Co‐IP

For Co‐IP experiments, chondrocytes were stimulated with IL‐1β for 24 h, then treated with the piezoelectric hydrogel combined with ultrasound for 3 days. Then, they were lysed in RIPA buffer containing protease/phosphatase inhibitors, and incubated with anti‐GATD3A antibody or normal IgG at 4°C overnight. Protein A/G magnetic beads were added and incubated at 4°C for 4 h. The complexes were eluted and subjected to Western blotting for the detection of TFAM and GAPDH.

### 4.24 Experimental OA Model

Twelve‐week‐old male C57BL/6 mice were used for destabilization of the medial meniscus (DMM) surgery or sham surgery on the right knee, as described previously [44]. In brief, for DMM surgery, mice were anesthetized by isoflurane inhalation, and the skin and joint capsule were incised. The joint cavity was accessed from the less fatty part of the joint capsule using microsurgical instruments (Figure S20). The medial meniscotibial ligament was transected to destabilize the meniscus, allowing it to displace medially. For the sham surgery, the skin and joint capsule were opened in the same way, but no further damage was inflicted. The incisions in the joint capsule and skin would be sutured layer by layer. All operations were performed under sterile conditions. During the observation period, if the mice showed discomfort, they were humanely euthanized. IA injections were started at 1 week post‐DMM and administered once per week thereafter until the endpoint (4 or 8 weeks). A 1 cm diameter probe was placed 2 mm above the sample (or on the shaved skin over the knee joint) with coupling gel. Ultrasound parameters were as follows: 1 MHz, 2.5 W/cm^2^, 5 min per session, once every other day. All mice were housed at the Experimental Animal Center of Hunan University. The experimental environment consisted of a 12 h light/dark cycle, with a temperature of 17–24°C, humidity of 70%, and the experimental subjects had free access to food and water. The subjects were euthanized by CO_2_. The relevant experimental protocol has been approved by the Animal Experiment Ethics Committee of Hunan University (Approval No: HNU‐IACUC‐2024‐114).

### 4.25 Micro‐CT

The knee joints were scanned using micro‐computed tomography (Micro‐CT) to analyze the subchondral bone microstructure of the tibial plateau. Parameters analyzed included bone volume fraction (BV/TV), trabecular thickness (Tb.Th), trabecular number (Tb.N), and trabecular separation (Tb.Sp). Three‐dimensional (3D) reconstruction was performed to visualize osteophyte formation.

### 4.26 Human Samples

After obtaining the approval from the Clinical Research Ethics Committee of the Third Affiliated Hospital of Southern Medical University (2025‐ER‐020), tibial plateau samples were collected from patients undergoing total knee arthroplasty (TKA) (5 male patients, aged between 50 and 80 years). After decalcification treatment, the samples were subjected to histological analysis. The hydrogels were first prepared and allowed to solidify directly in the culture wells. After gelation, cartilage explants (or chondrocytes) were placed on top of the solidified hydrogels. The co‐culture system was then maintained in DMEM containing 10% FBS at 37°C in a 5% CO_2_ incubator. Ultrasound stimulation was applied after the co‐culture was established.

### 4.27 Transcriptome Analysis

To profile the transcriptomic changes induced by the piezoelectric hydrogel, primary mouse chondrocytes were divided into two groups. The IL‐1β group was treated with 30 ng/mL IL‐1β for 24 h. The CMB Gel group received the same IL‐1β stimulation, followed by co‐culture with Cap‐CM@BTO/Gel and ultrasound stimulation for 3 days. After treatment, cells were harvested, and total RNA was extracted using TRIzol reagent (Invitrogen, USA) according to the manufacturer's instructions. These RNA samples were stored at ‐80°C before sequencing. The total RNA extraction, purification, library construction, and sequencing were all completed by Beijing Qingke Biotechnology Co. Ltd.

### 4.28 Statistical Analysis

All experiments in this study were conducted at least three times. All data are expressed as mean ± standard deviation (SD) and charted using Origin 2018 software (Origin Lab Corporation, USA). Analysis was performed using the Student *t* test (unpaired and two‐tailed), one‐way or two‐way ANOVA, and then Tukey post hoc analysis. For two groups, unpaired *t*‐test with Bonferroni correction when appropriate. *p* value < 0.05 was considered statistically significant.

## Author Contributions

**Hui Zheng** and **Pengfei Yan** conceived the project and designed and performed most of the experiments, discussed the results, and co‐wrote the manuscript. Pengliu and ChangYan an assisted in histology and imaging analysis. **Mengqi Zhao** contributed to the hot plate test and micro–computed tomography data acquisition. **Zuyong Wang**, **Rongkai Zhang**, **Chao Ma**, and Sweehin Teoh provided suggestions on project design and data presentation. Sweehin Teoh supervised all experiments and revised the manuscript. Xi Cui made significant contributions to the revision of the manuscript and provided essential editorial work that improved the final version.

## Funding

This work was supported by the National Natural Science Foundation of China: Z202301482550 and the Science and Technology Innovation Program of Hunan Province (2024RC9004).

## Conflicts of Interest

The authors declare no conflicts of interest.

## Supporting information




**Supporting File**: advs76140‐sup‐0001‐SuppMat.pdf.

## Data Availability

The data that supports the findings of this study are available in the supplementary material of this article.
