# Supplementary material for: Ultrasound‐Activatable Piezoelectric Hydrogel Reprograms Mitochondrial Epigenetics for Osteoarthritis Therapy via the mTOR/GATD3A Axis
Source: Adv Sci (Weinh). 2026 Jun 16:e76140. Online ahead of print. doi: 10.1002/advs.76140 (PMC13337106; doi:10.1002/advs.76140)
Supplement: Supplementary file 1 — Supporting File: advs76140‐sup‐0001‐SuppMat.pdf. [file ADVS-9999-e76140-s001.pdf]

Supplementary Materials for

Ultrasound-Activatable Piezoelectric Hydrogel Reprograms Mitochondrial Epigenetics for Osteoarthritis Therapy via the mTOR/GATD3A Axis

Hui Zheng [1]†, Pengfei Yan [1]†et al.

* Corresponding Author: Swee Hin Teoh. PhD, E-mail: Teohsh@hnu.edu.cn

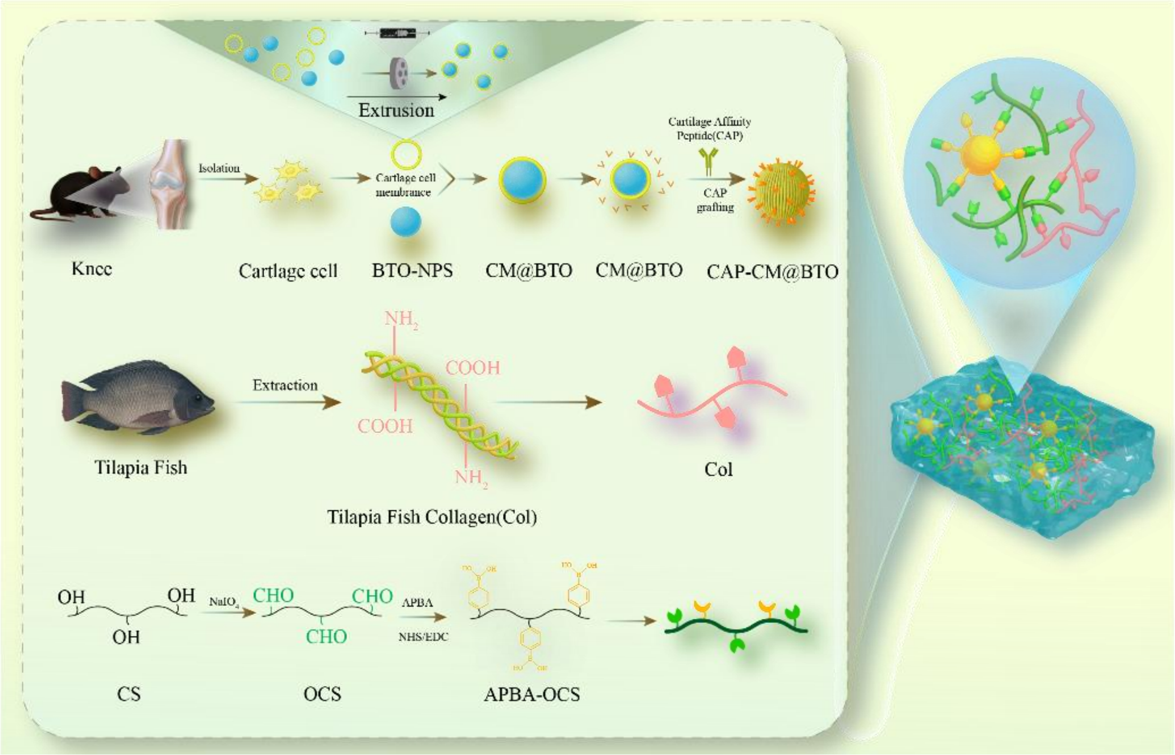

Fig.S1: The design strategy of biomimetic piezoelectric hydrogel.

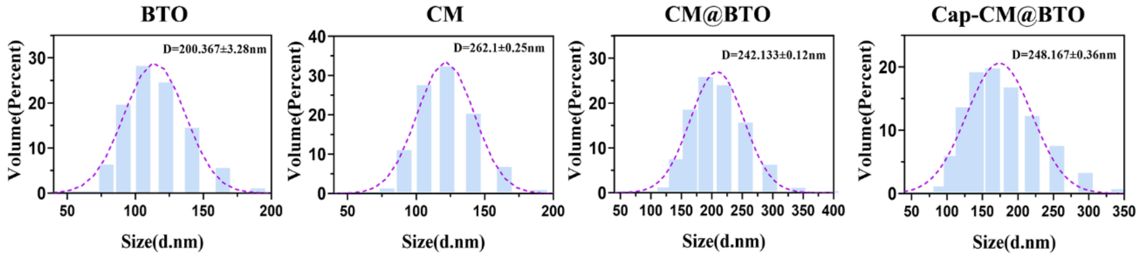

Fig.S2: Hydrodynamic diameters of BTO-NPs, CM vesicles, CM@BTO and Cap-CM@BTO measured by dynamic light scattering (DLS).

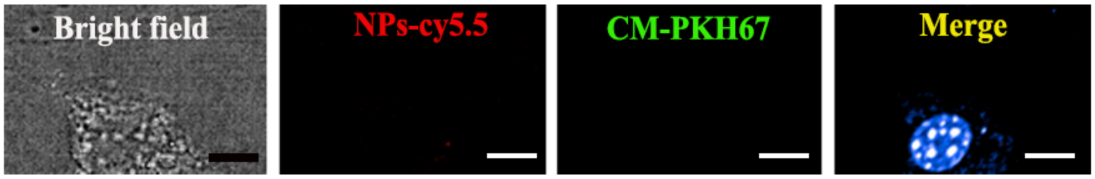

Fig.S3: Representative confocal microscopy images of mouse chondrocytes treated with CM@NPs.
The BTO core was labeled with Cy5.5 (red), the CM shell with PKH67 (green), and the nuclei with DAPI (blue). Scale bar, 20 μm.

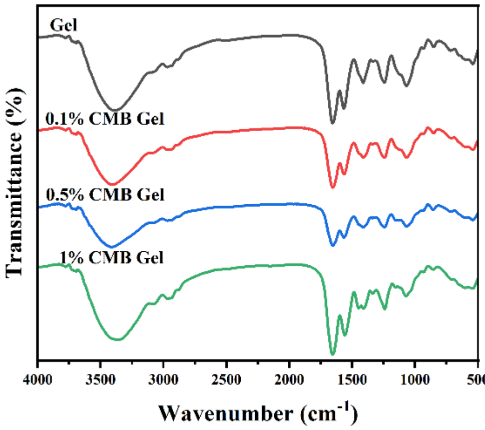

Fig.S4: FT-IR spectra of Gel,0.1% CMB Gel, 0.5% CMB Gel and 1% CMB Gel hydrogels.

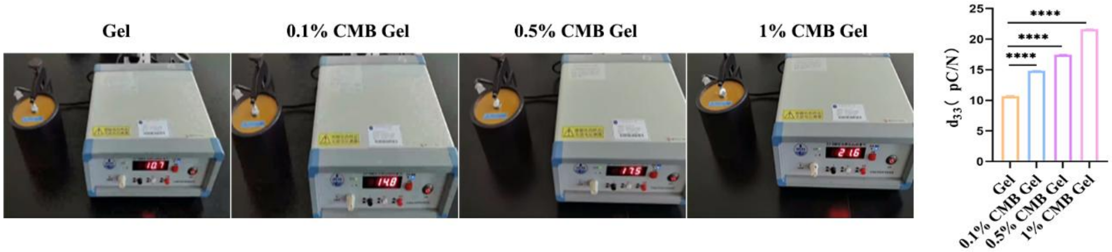

Fig.S5: Piezoelectric constants of different hydrogels.
Statistical analysis was performed using one-way analysis of variance (ANOVA) combined with Tukey's post hoc test. n=3, $*P < 0.05$, $***P < 0.001$. ns, no significant difference.

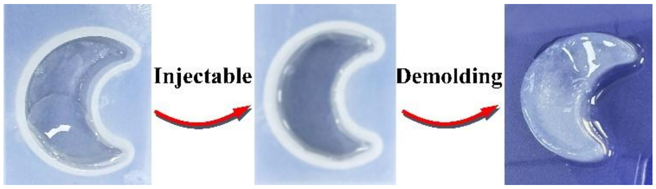

Fig.S6: The injectability of piezoelectric hydrogels.

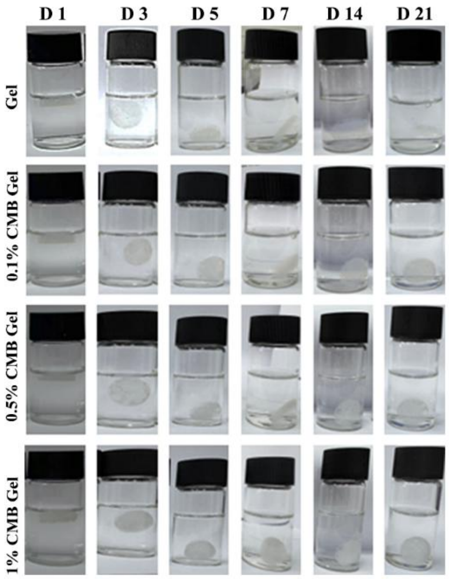

Fig.S7: ***In vitro* degradation.**
The hydrogel was placed in a phosphate buffer saline solution at 37℃, which was prepared with 60 micrograms per milliliter of type I collagenase, and then left to be subjected to in vitro degradation performance measurement.

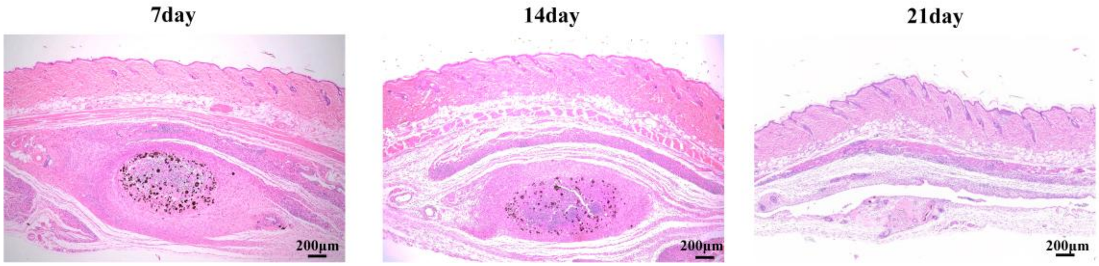

Fig.S8: The degradation of hydrogels *in vivo.* Scale bar, 200μm.

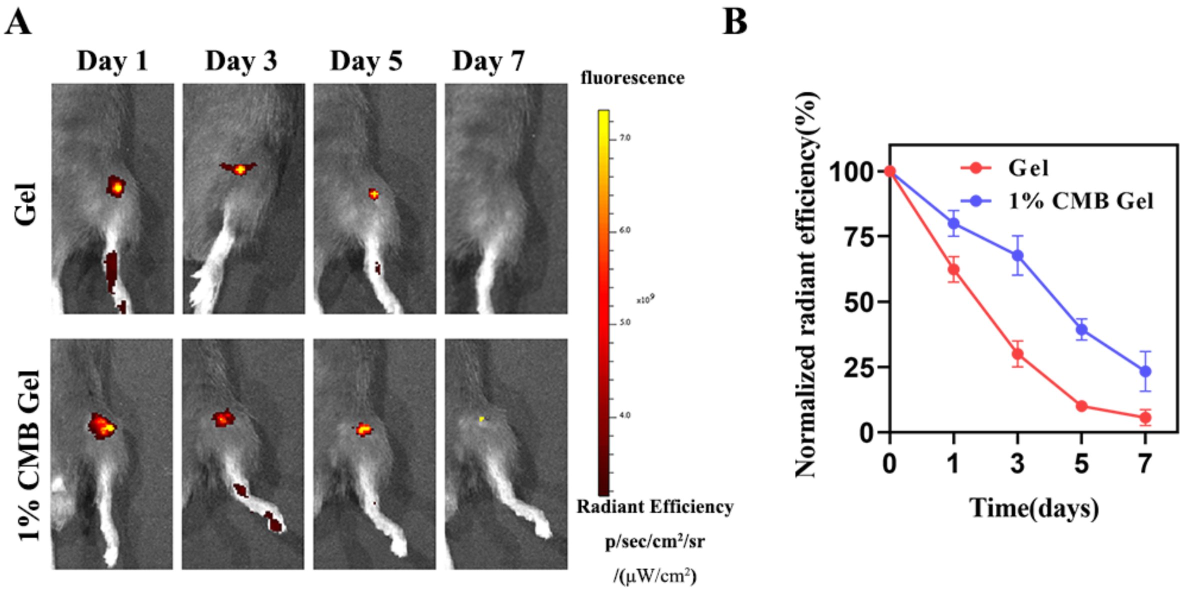

Fig.S9: *In vivo* retention of hydrogels in mouse knee joints.
(A) Representative IVIS images of Cy5.5-labeled Gel and CMB Gel at indicated time points. **(B)** Quantification of standardized fluorescence radiation efficiency showing significantly prolonged retention of CMB Gel compared to Gel ($n = 3$, mean ± SD, ***$p < 0.001$ vs. Gel).

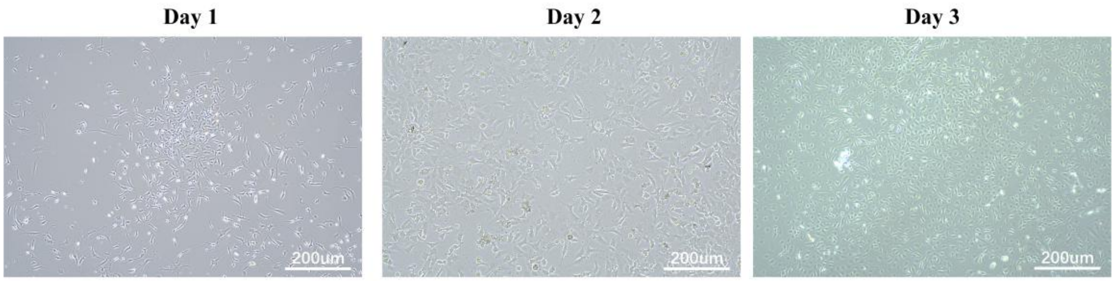

Fig.S10: Representative images of primary chondrocyte from mice. Scale bar, 200μm.

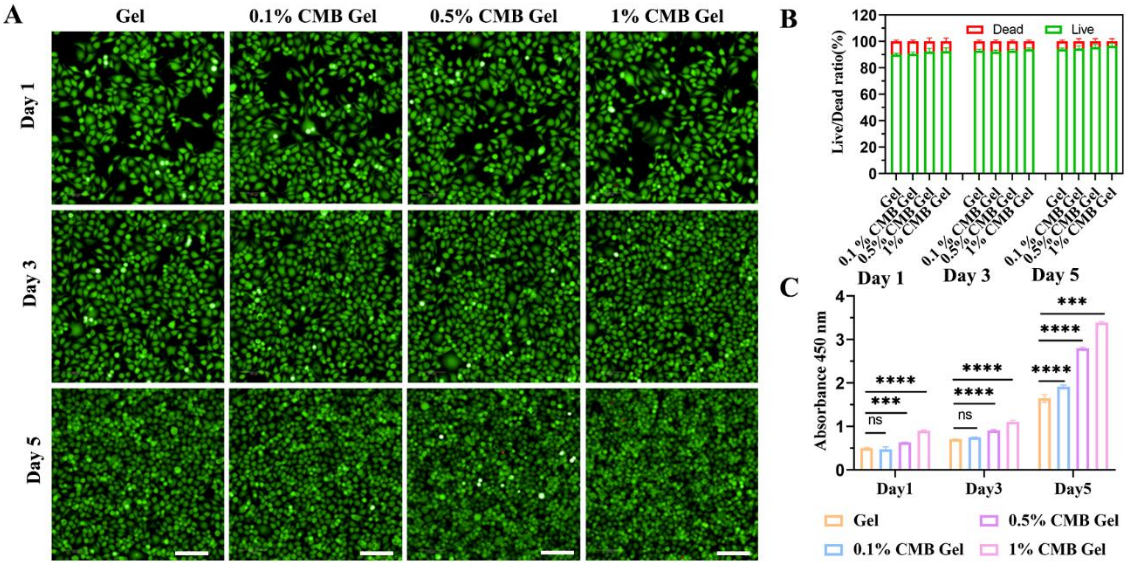

Fig.S11: Evaluation of the biocompatibility of the hydrogel on primary chondrocytes.
(A) The CLSM images show the live/dead staining of primary mouse chondrocytes on different hydrogels on days 1, 3, and 5. Scale bar, 200 µm. (B) Quantification of the live/dead cell percentages corresponding to (A). (C) The proliferation of primary chondrocytes cultured on different hydrogels was studied using the CCK-8 assay. Statistical analysis was performed using one-way analysis of variance (ANOVA) combined with Tukey's post hoc test. n=5, *P < 0.05, ***P < 0.001. ns, no significant difference.

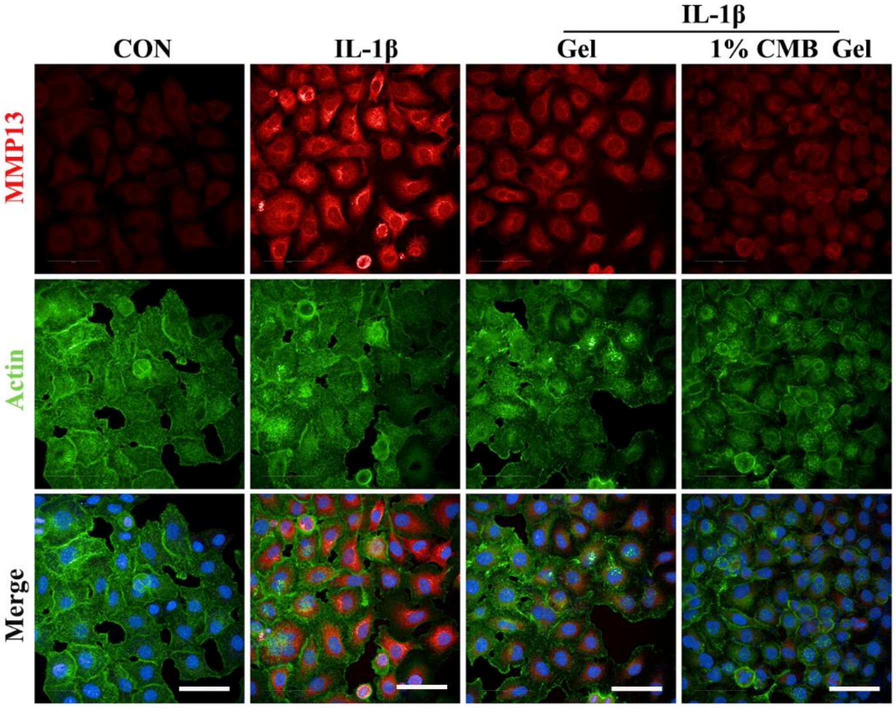

Fig.S12: Representative triple-immunofluorescence images: MMP13 (red), F-actin (green, stained with FITC-phalloidin), and cell nuclei (blue, DAPI). Scale bar , 50 µm. Treatment groups are

defined as follows: CON, untreated chondrocytes; IL-1β, cells treated with 5 ng/mL IL-1β for 24 h; Gel, IL-1β plus hydrogel without nanoparticles; CMB Gel, IL-1β plus 1% Cap-CM@BTO/Gel with ultrasound (1 MHz, 2.5 W/cm², 5 min/session, 3 sessions/day).

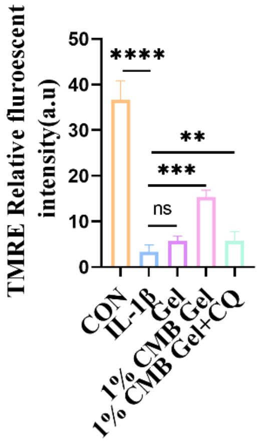

Fig.S13: Quantification of TMRE fluorescence intensity.
Statistical analysis was performed using one-way analysis of variance (ANOVA) combined with Tukey's post hoc test. n=5, *P < 0.05, ***P < 0.001. ns, no significant difference.

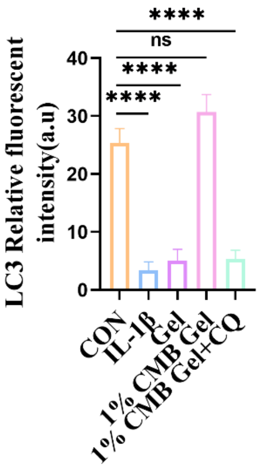

Fig.S14:LC3 puncta per cell were quantified from confocal images.
Statistical analysis was performed using one-way analysis of variance (ANOVA) combined with Tukey's post hoc test. n=20, *P < 0.05, ***P < 0.001. ns, no significant difference.

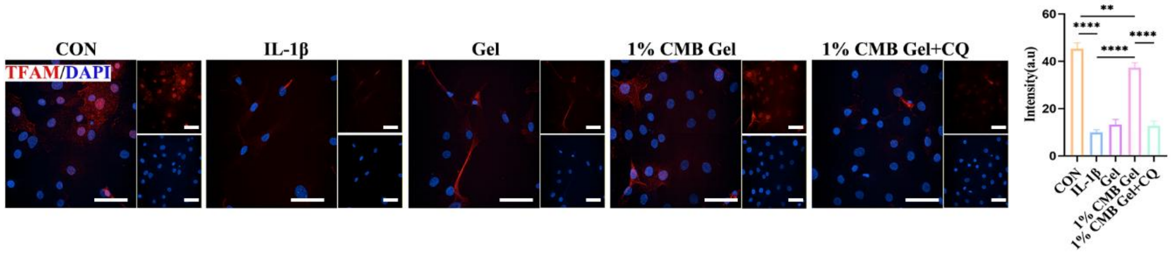

Fig.S15: Representative image of TFAM (red) immunofluorescence staining in chondrocytes. Scale bar, 50μm. Statistical analysis was performed using one-way analysis of variance (ANOVA) combined with Tukey's post hoc test. n=5, *P < 0.05, ***P < 0.001. ns, no significant difference.

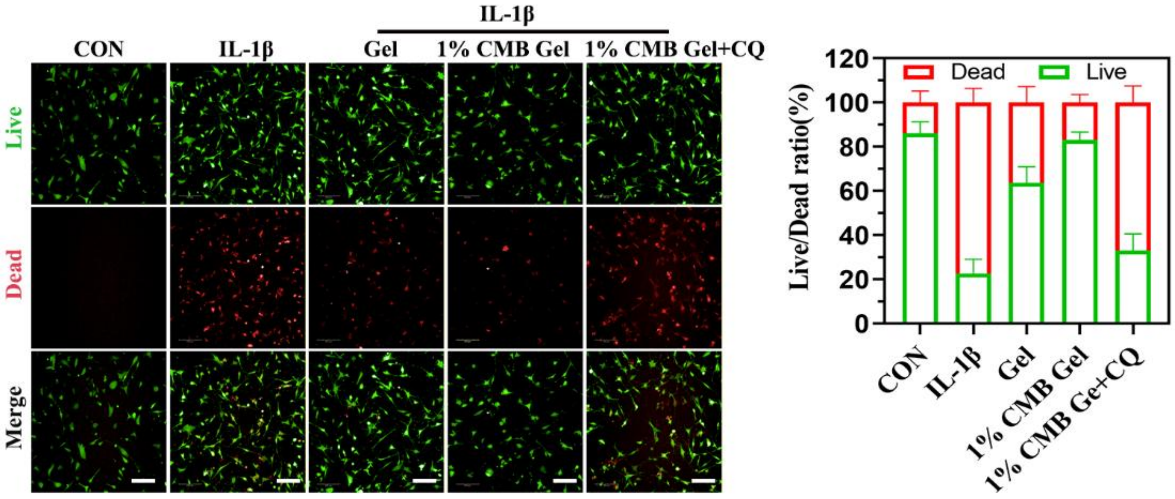

Fig.S16: Representative image of live/dead immunofluorescence staining of chondrocytes in different groups. Scale bar, 200μm.Statistical analysis was performed using one-way analysis of variance (ANOVA) combined with Tukey's post hoc test. n=5, *P < 0.05, ***P < 0.001. ns, no significant difference.

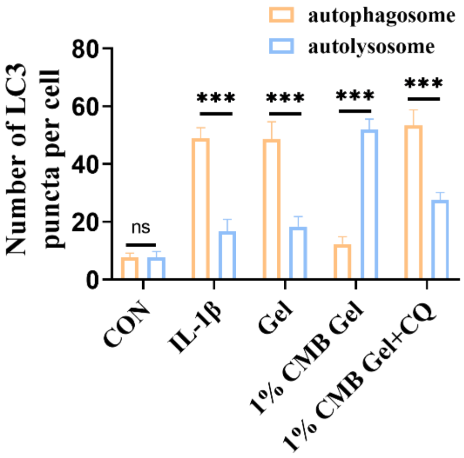

Fig.S17: After transfecting Chondrocytes cells with mCherry-GFP-LC3, subsequent experiments included IL-1β stimulation, ultrasound activation treatment, and assessment of autophagosomes (yellow) and autolysosomes (red) formation. Statistical analysis was performed using one-way analysis of variance (ANOVA) combined with Tukey's post hoc test. n=15, *P < 0.05, ***P < 0.001. ns, no significant difference.

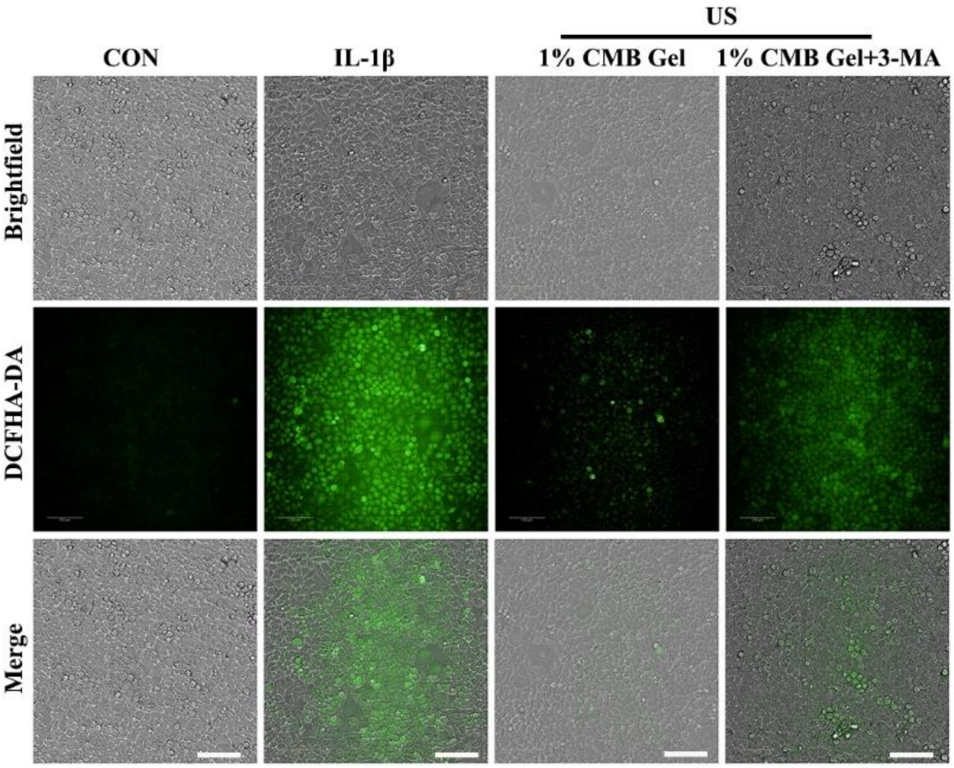

Fig.S18: Representative image of reactive oxygen species immunofluorescence staining in different groups. Scale bar, 200μm.

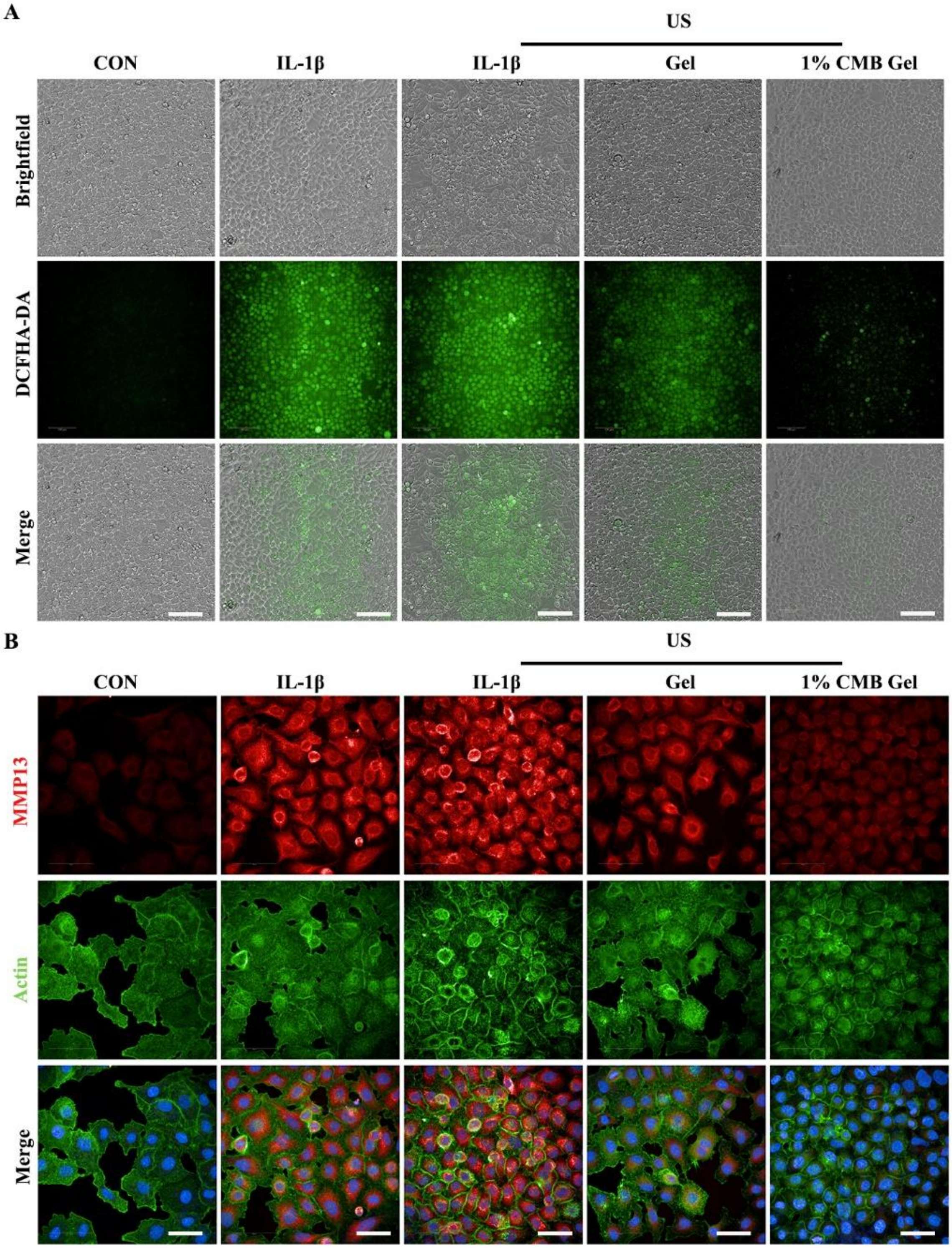

Fig.S19：(A)Representative fluorescence images of intracellular ROS detected by DCFH-DA (green). Scale bar, 200 μm.(B) Representative immunofluorescence images of MMP13 (red) with DAPI nuclear counterstaining (blue). Scale bar, 50 μm.

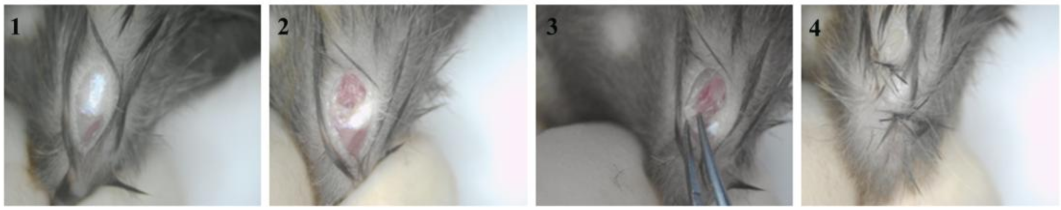

Fig.S20: Representative digital images of the surgical process of the mouse DMM model.

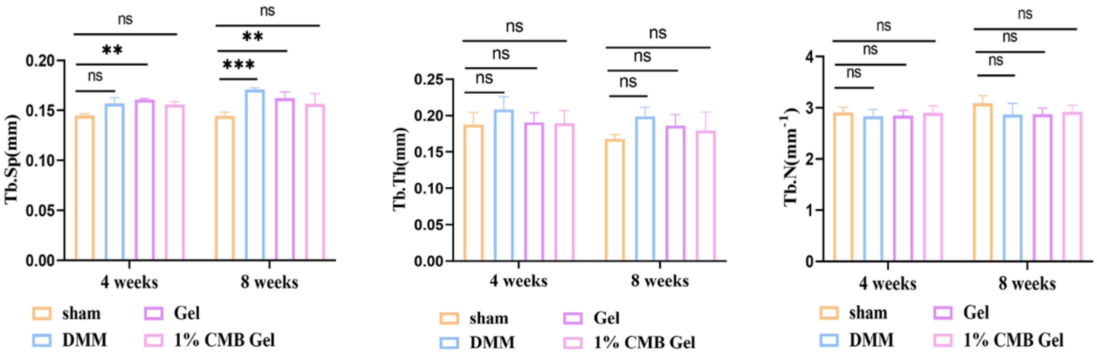

Fig.S21: Quantitative analysis of trabecular bone under cartilage at 4 and 8 weeks after surgery and injection among groups.

Statistical analysis was performed using one-way analysis of variance (ANOVA) combined with Tukey's post hoc test. n=6, $^*P < 0.05$, $^{***}P < 0.001$. ns, no significant difference.

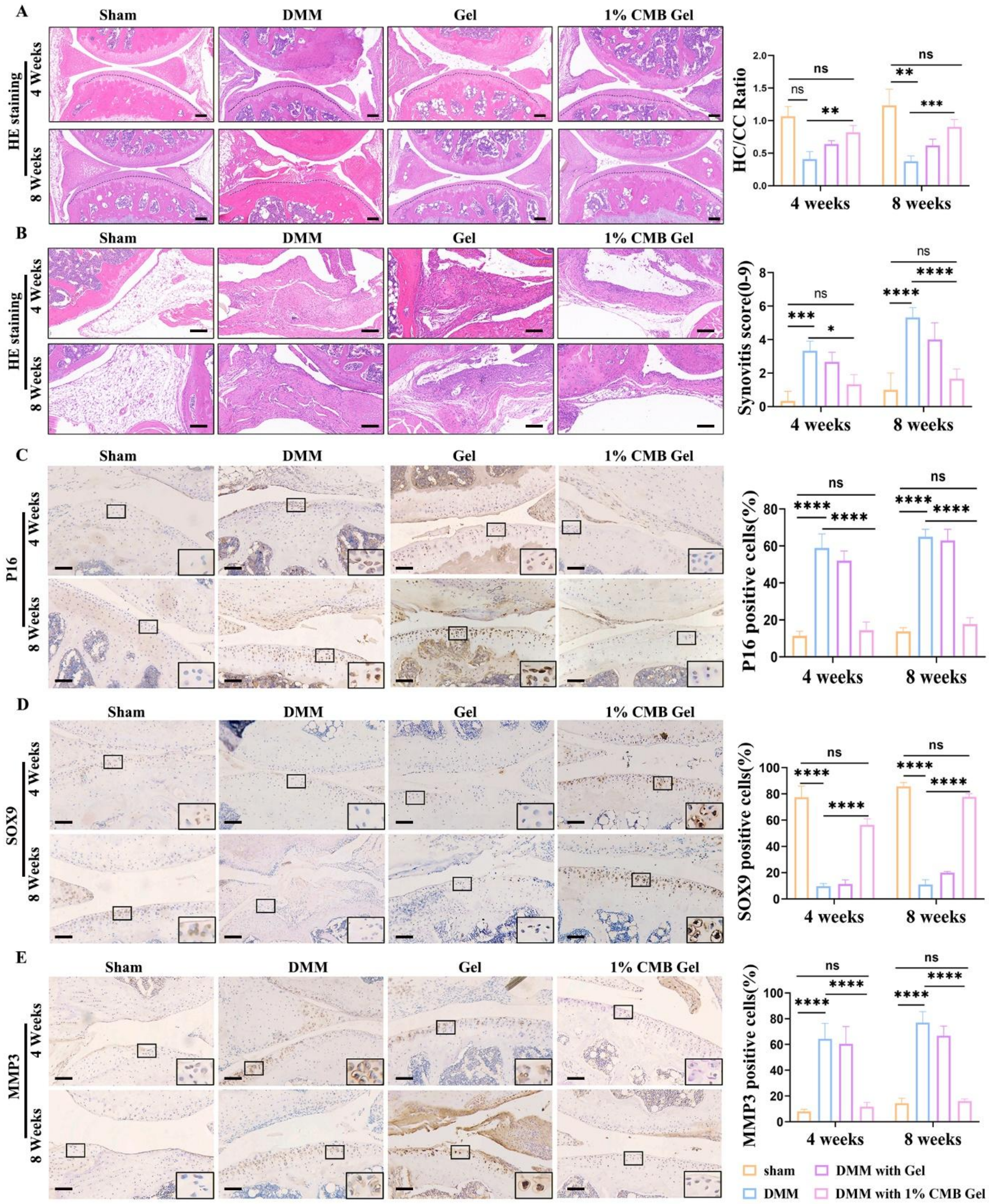

Fig.S22: (A) HE staining of the knee joints of each group was performed 4 weeks and 8 weeks after treatment. Scale bar:100μm.(B) After 4 weeks and 8 weeks of treatment, perform HE staining on the synovium of each group. Scale bar, 200 μm. (C, D, E) MMP3, SOX9 and P16 immunohistochemical staining of the knee joint cartilage of mice was conducted 4 weeks and 8 weeks after treatment. Scale bar,100μm. The data

are presented in the form of mean ± standard deviation. Statistical analysis was performed using one-way analysis of variance (ANOVA) combined with Tukey's post hoc test. n=6, *P < 0.05, ***P < 0.001. ns, no significant difference.

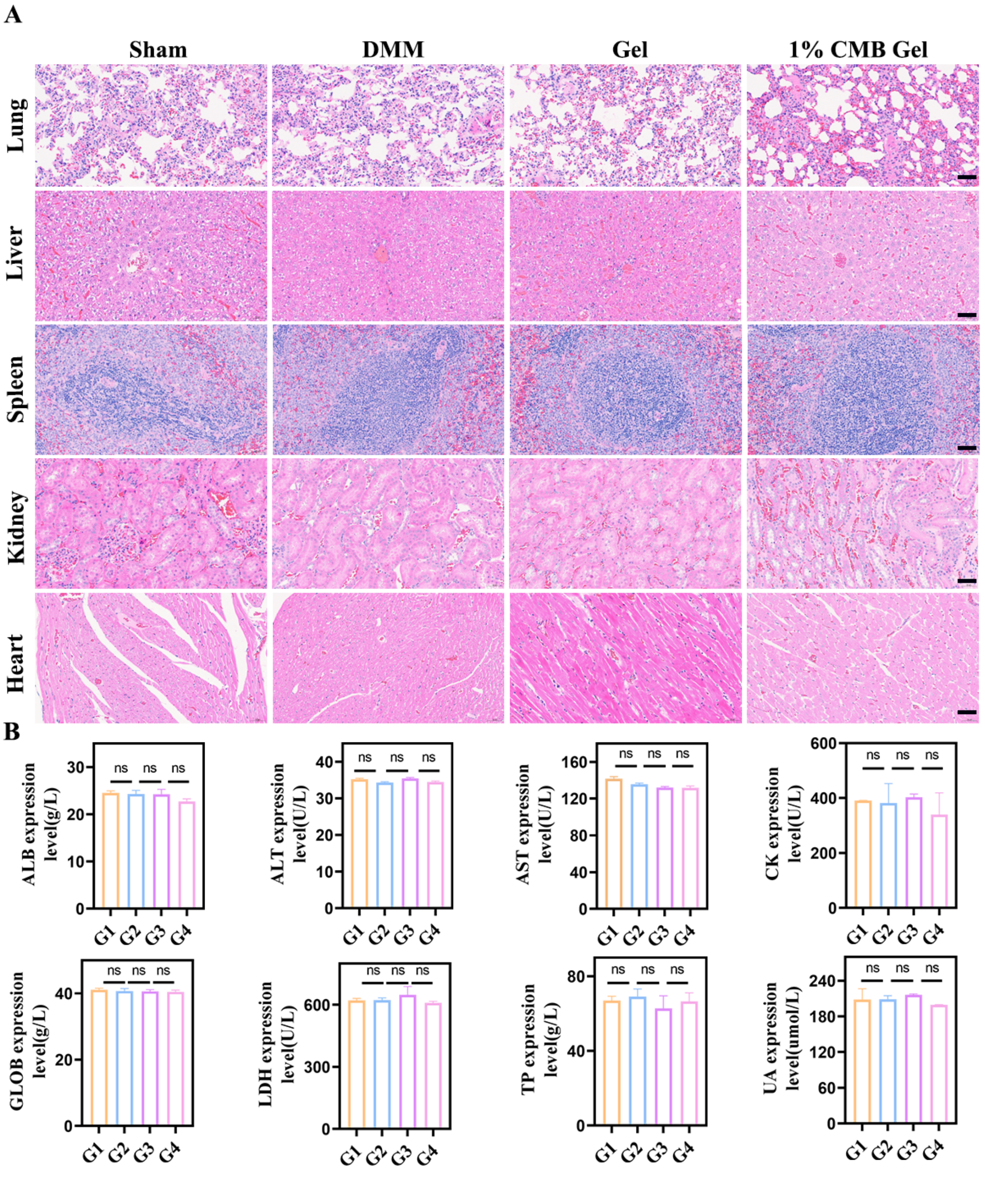

Fig.S23:1% CMB Gel Biocompatibility Analysis

(A) H&E staining images of major organs (heart, liver, spleen, lung, kidney). Scale bar, 50 μm. (B) Analysis of blood biochemical indicators: ALB, ALT, AST, CK, GLB, LDH, TP, UA; G1: sham group; G2: DMM group; G3: Gel group; G4: 1% CMB Gel group. Statistical analysis was performed using one-way analysis of variance (ANOVA) combined with Tukey's post hoc test. n=6*P < 0.05, ***P < 0.001. ns, no significant difference.

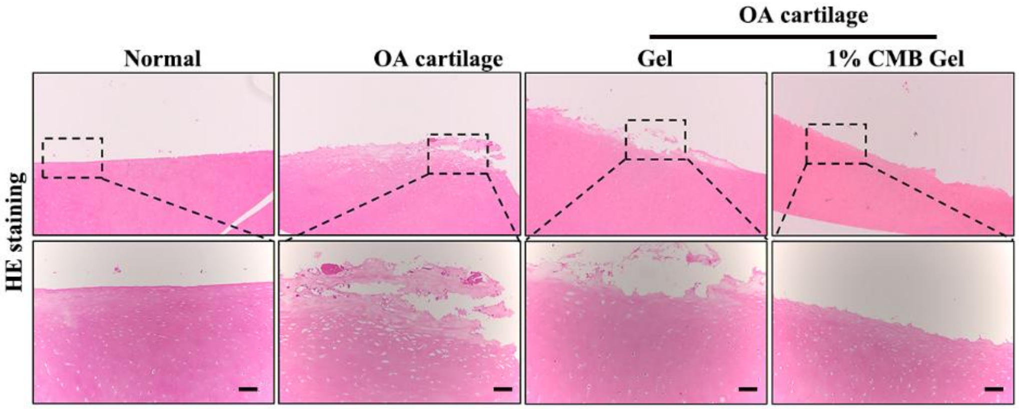

Fig S24: HE staining of different groups of cartilage samples. Scale bar, 200 μm.

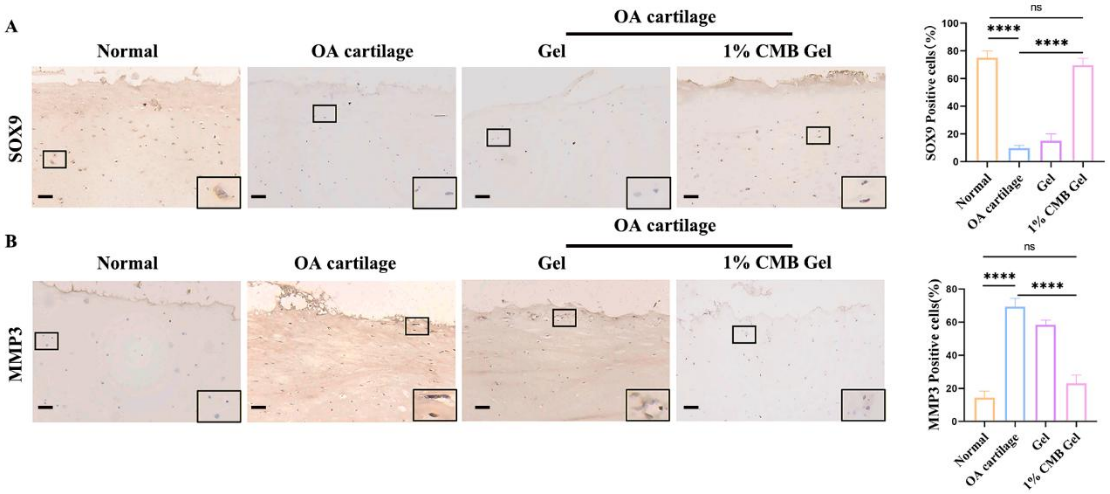

Fig.S25: (A, B) Immunohistochemical staining results of MMP3 and SOX9. Scale bar, 100 μm. Statistical analysis was performed using one-way analysis of variance (ANOVA) combined with Tukey's post hoc test. n=5, $*P < 0.05$, $***P < 0.001$. ns, no significant difference.

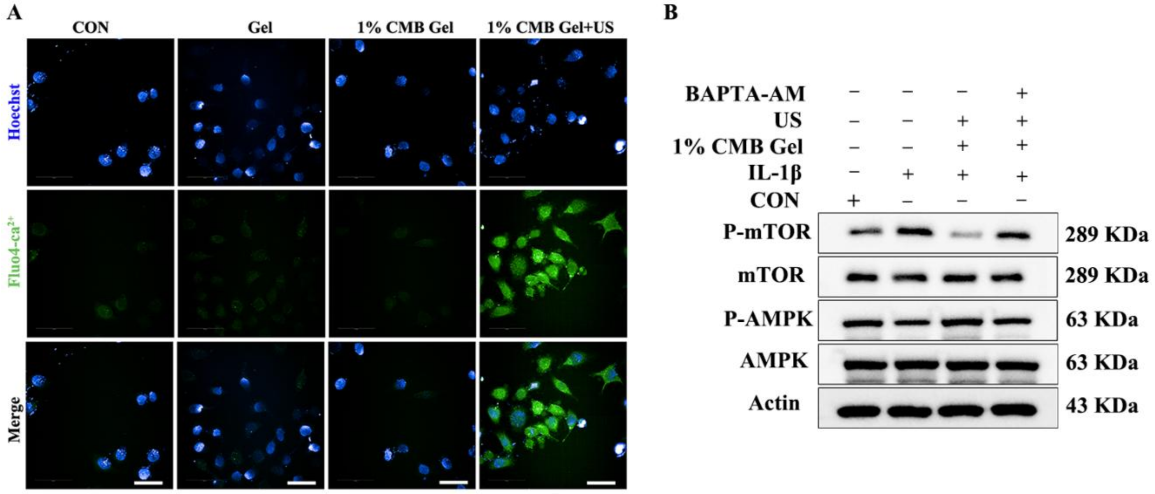

Fig.S26: (A) Representative fluorescence images of intracellular Ca^{2+} detected by Fluo-4 AM (green). Scale bar, 50 μm. (B)Representative Western blot images of p-AMPK, AMPK, p-mTOR, and mTOR. GAPDH was used as a loading control.

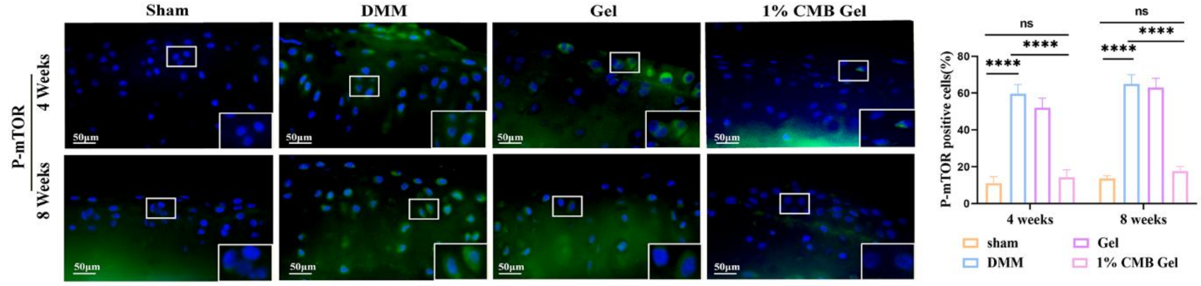

Fig.S27: After 4 weeks and 8 weeks of treatment, immunofluorescence staining for P-mTOR was performed on the knee joint cartilage of the mice. scale bar,50μm. Statistical analysis was performed using one-way analysis of variance (ANOVA) combined with Tukey's post hoc test. n=6, *P < 0.05, ***P < 0.001. ns, no significant difference.

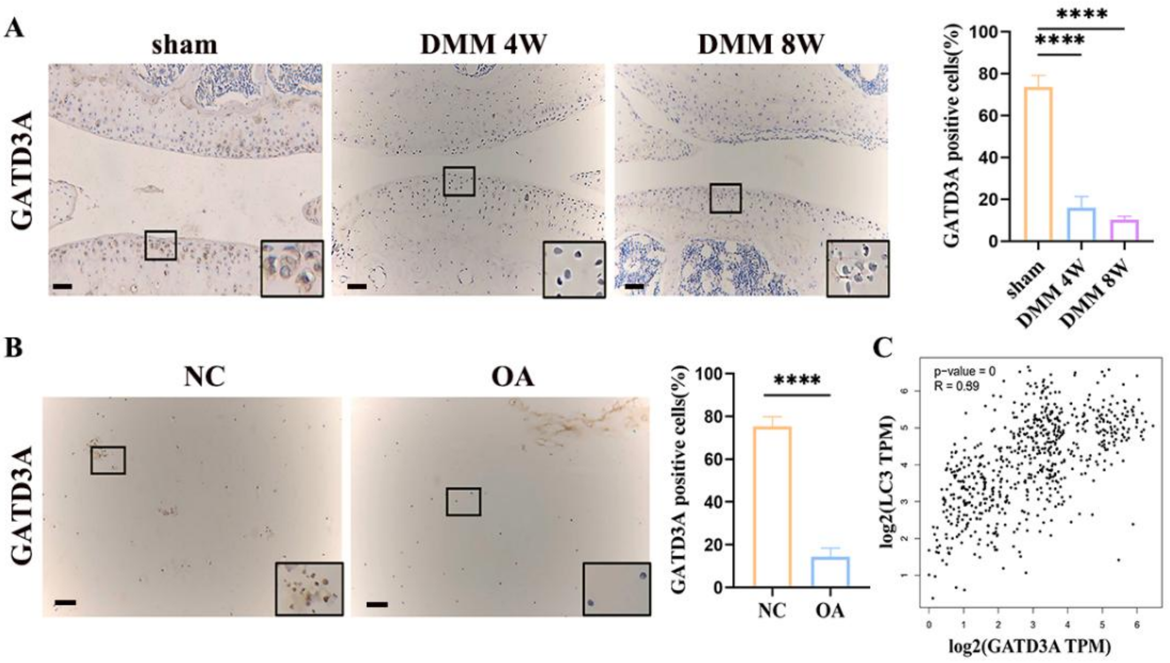

Fig.S28: (A)Immunohistochemical staining of GATD3A on the knee joints of mice in the control group and at 4, 8 weeks after DMM. Scale bar, 100μm.(B) Immunohistochemical staining of GATD3A in human osteoarthritis chondrocytes. Scale bar, 100μm.(C) Analysis of the correlation between GATD3A and LC3 using the GEPIA database. Statistical analysis was performed using one-way analysis of variance (ANOVA) combined with Tukey's post hoc test. n=6, *P < 0.05, ***P < 0.001. ns, no significant difference.

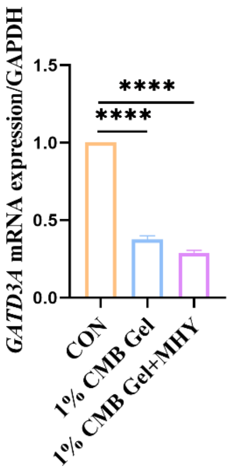

Fig.S29: RT-qPCR analysis of GATD3A level in chondrocytes using ultrasound combined with piezoelectric hydrogel and MHY1485 (mTOR activator) (with or without IL-1b stimulation). Statistical analysis was performed using one-way analysis of variance (ANOVA) combined with Tukey's post hoc test. n=3, *P < 0.05, ***P < 0.001. ns, no significant difference.

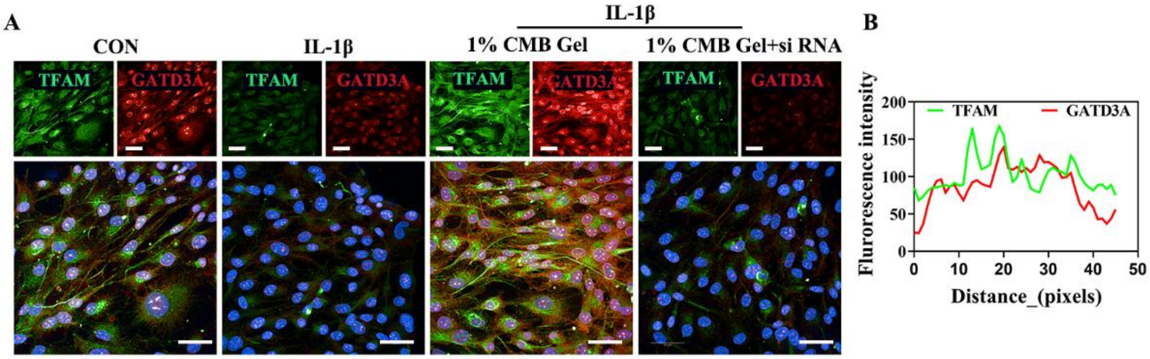

Fig.S30: (A) Representative immunofluorescence image showing the subcellular localization of GATD3A (red) and TFAM (green) in chondrocytes. Scale bar, 50μm. (B) Co-localization analysis of TFAM and GATD3A by ImageJ Plot profile.

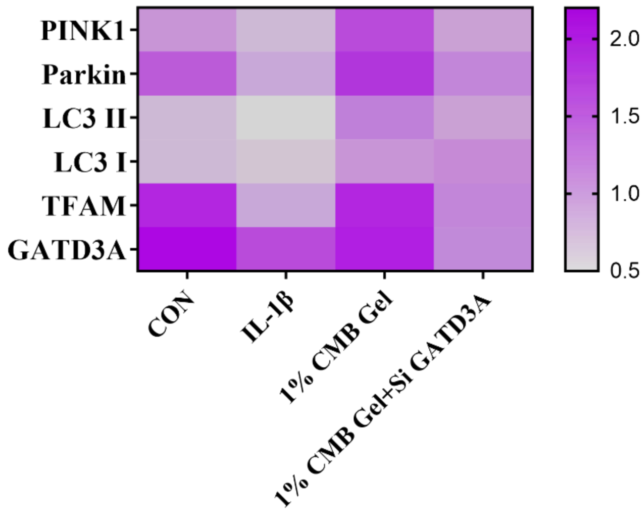

Fig.S31: Quantitative analysis of Western blot gray value.

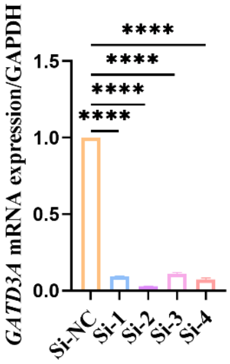

Fig.S32. Afte transfection with siRNA, the level of GATD3A in chondrocytes was analyzed by RT-qPCR. Statistical analysis was performed using one-way analysis of

variance (ANOVA) combined with Tukey's post hoc test. n=3, *P < 0.05, ***P < 0.001. ns, no significant difference.

Table.S1. Primer sequences of real-time PCR analysis.

Gene	Forward (5'-3')	Reverse (3'-5)
GAPDH	CCTCGTCCCGTAGACAAAATG	TGAGGTCAATGAAGGGGTCGT
MMP13	GTTTCTTTATGGTCCAGGCGAT	GACTGGTAATGGCATCAAGGGA
COL2	GGAAGAGCGGAGACTACTGGATT	GCTCTTGCTGCTCCACCAGTT
P16	GGACATCAAGACATCGTGCG	TTGAGCTGAAGCTATGCCCG
P21	CTTGTCGCTGTCTTGCACTCTG	GAAATCTGTCAGGCTGGTCTGC
P53	CCCTCTGAGCCAGGAGACATT	CCCAGGTGGAAGCCATAGTTG
SOX9	CAGTACCCGCATCTGCACAAC	AGCGCCTTGAAGATAGCATTAG
Adamt5	CCTACCGCCATTGTAATAACCC	TCAGTCCCATCCGTAACCTTTG
GATD3A	AAAGTGATCAAAGGTGTGGAGGT	CTTTGATGGCTTCCGCAGTT
mTOR	GCAACAACCTCCAGGATACACTAA	ACGGGCTGTGGTGGTAGACTTA

Table.S2. The siGATD3A sequences.

Gene	Forward (5'-3')	Reverse (3'-5)
**Si-1**	GGCAGAGUCAGCAAGGAUUTT	AAUCCUUGCUGACUCUGCCTT
**Si-2**	GUGUGACCGAAGCUCAUGUTT	ACAUGAGCUUCGGUCACACTT
**Si-3**	GGAAAGGACUGCAAGGUUATT	UAACCUUGCAGUCCUUUCCTT
**Si-4**	CCAAAGUGAUCAAAGGUGUTT	ACACCUUUGAUCACUUUGGTT

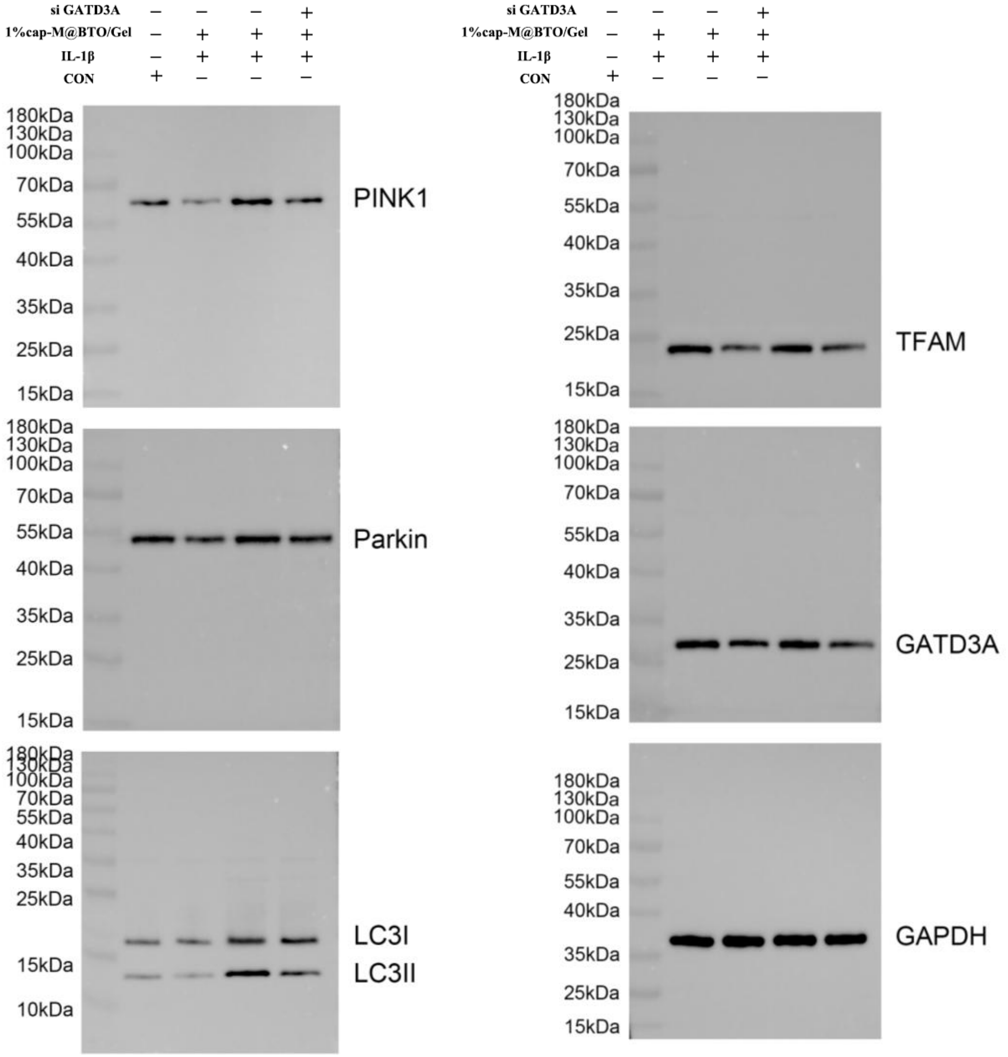
si GATD3A
1%cap-M@BTO/Gel
IL-1β
CON
PINK1
Parkin
LC3I
LC3II
TFAM
GATD3A
GAPDH
180kDa
130kDa
100kDa
70kDa
55kDa
40kDa
35kDa
25kDa
15kDa
10kDa

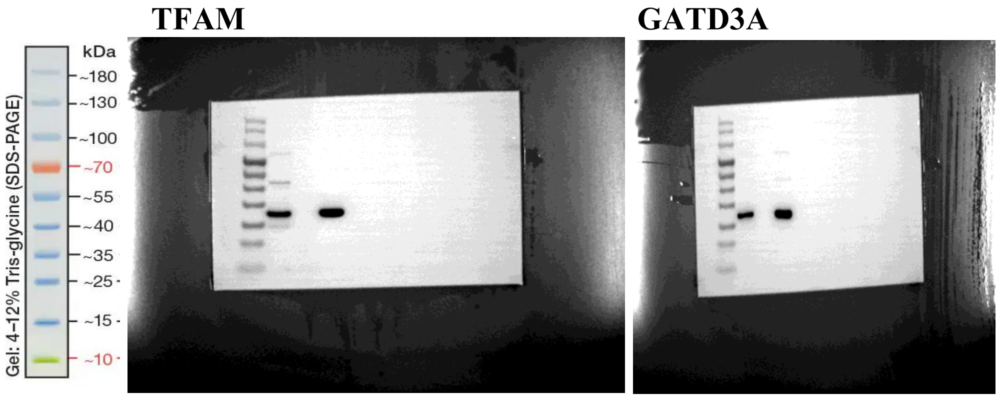
TFAM
GATD3A
kDa
~180
~130
~100
~70
~55
~40
~35
~25
~15
~10
Gel: 4–12% Tris-glycine (SDS-PAGE)
